# Development and validation of a Luminex assay for detection of a predictive biomarker for PROSTVAC-VF therapy

**DOI:** 10.1371/journal.pone.0182739

**Published:** 2017-08-03

**Authors:** Julie L. Lucas, Erin A. Tacheny, Allison Ferris, Michelle Galusha, Apurva K. Srivastava, Aniruddha Ganguly, P. Mickey Williams, Michael C. Sachs, Magdalena Thurin, James V. Tricoli, Winnie Ricker, Jeffrey C. Gildersleeve

**Affiliations:** 1 MRIGlobal, Gaithersburg, Maryland, United States of America; 2 Pharmacodynamics Biomarker Program, Applied/Developmental Research Directorate Frederick National Laboratory for Cancer Research, Leidos Biomedical Research, Inc., Frederick, Maryland, United States of America; 3 Cancer Diagnosis Program, Division of Cancer Treatment and Diagnosis, National Cancer Institute, National Institutes of Health, Bethesda, Maryland, United States of America; 4 Molecular Characterization and Clinical Assay Development Laboratory, Frederick National Laboratory for Cancer Research, Frederick, Maryland, United States of America; 5 Biostatistics Branch, Biometric Research Program, NCI, NIH, Bethesda, Maryland, United States of America; 6 Diagnostic Biomarkers and Technology Branch, Cancer Diagnosis Program Division of Cancer Treatment and Diagnosis, National Cancer Institute, Rockville, Maryland, United States of America; 7 Information Management Services, Inc., Rockville, Maryland, United States of America; 8 Chemical Biology Laboratory, Center for Cancer Research, National Cancer Institute, National Institutes of Health, Frederick, Maryland, United States of America; University of Minnesota Hormel Institute, UNITED STATES

## Abstract

Cancer therapies can provide substantially improved survival in some patients while other seemingly similar patients receive little or no benefit. Strategies to identify patients likely to respond well to a given therapy could significantly improve health care outcomes by maximizing clinical benefits while reducing toxicities and adverse effects. Using a glycan microarray assay, we recently reported that pretreatment serum levels of IgM specific to blood group A trisaccharide (BG-A_tri_) correlate positively with overall survival of cancer patients on PROSTVAC-VF therapy. The results suggested anti-BG-A_tri_ IgM measured prior to treatment could serve as a biomarker for identifying patients likely to benefit from PROSTVAC-VF. For continued development and clinical application of serum IgM specific to BG-A_tri_ as a predictive biomarker, a clinical assay was needed. In this study, we developed and validated a Luminex-based clinical assay for measuring serum IgM specific to BG-A_tri_. IgM levels were measured with the Luminex assay and compared to levels measured using the microarray for 126 healthy individuals and 77 prostate cancer patients. This assay provided reproducible and consistent results with low %CVs, and tolerance ranges were established for the assay. IgM levels measured using the Luminex assay were found to be highly correlated to the microarray results with R values of 0.93–0.95. This assay is a Laboratory Developed Test (LDT) and is suitable for evaluating thousands of serum samples in CLIA certified laboratories that have validated the assay. In addition, the study demonstrates that discoveries made using neoglycoprotein-based microarrays can be readily migrated to a clinical assay.

## Introduction

Cancer vaccines and other immunotherapies exploit the power of the immune system to target and eliminate cancer cells within a patient’s body. [1] Immune-based therapies can produce long lasting clinical responses, and many have received FDA approval or are in late stage clinical trials. While these therapies are transforming cancer care, some patients have remarkable responses while others have no apparent clinical benefit. Methods to pre-select patients that are likely to respond favorably to a given therapy could significantly improve patient outcomes while minimizing adverse effects. [1]

PROSTVAC-VF is a poxvirus-based cancer vaccine for the treatment of advanced prostate cancer. [2–5] This vaccine induces immune responses to prostate-specific antigen (PSA) using genetically modified vaccinia and fowlpox encoding PSA and 3 costimulatory molecules (LFA-3, B7.1, and ICAM-1). In two phase II clinical trials, PROSTVAC-VF was associated with an increase in median survival of 8 to 9 months, and it is currently in phase III clinical trials. [3,4] While promising, not all patients experience improved survival and therefore strategies to guide targeted therapy to patients likely to respond favorably would be advantageous.

In a previous study, a carbohydrate antigen microarray (also referred to as a “glycan array” or “glyco-antigen microarray” [6–10]) was used to profile patient serum antibody levels to characterize immune responses in cancer patients and identify potentially diagnostic biomarkers predictive to treatment. IgM serum antibodies that bind blood group A trisaccharide (anti-BG-A_tri_ IgM) measured prior to treatment were found to correlate significantly with overall survival in patients from two separate Phase II clinical trials. [11] Blood group A is a trisaccharide composed of the sequence GalNAcα1-3(Fucα1–2)Gal. It is one of the antigens that defines ABO blood type and is best known for its presence on the surface of red blood cells of individuals with blood type A or AB. We have found that it is also present on the surface of the poxviruses, and we have postulated that antibody binding to BG-A on the poxvirus provides an adjuvant effect which enhances immune responses. [11] The results suggested that anti-BG-A_tri_ IgM measured prior to treatment could be used for screening to identify patients likely to respond favorably to PROSTVAC-VF therapy. While serum antibody levels to blood group A antigen are typically high in individuals with blood type O or B and low in individuals with blood type A or AB, blood type is not a reliable surrogate for the presence of serum anti-BG-A_tri_ IgM antibodies. In particular, correlations with blood type are weaker for IgM than IgG antibodies, and some patients with type A or AB blood have relatively high levels of anti-BG-A_tri_ IgM. [12]

Although the glyco-antigen microarray is well suited for discovery, it is not an ideal platform for a clinical assay. Microarrays require specialized robotic equipment for production, are expensive, and can be technically demanding to perform. For continued development (e.g. analysis of the ~1200 patients in the Phase III trial) and clinical application of serum anti-BG-A_tri_ IgM as a predictive biomarker, a standardized, highly reproducible, efficient, and cost effective assay that would meet the rigorous performance standards of a clinical assay was warranted. Conversion of a glyco-antigen microarray assay to a clinical assay has not previously been reported. In this study, we describe the development and validation of a Luminex bead-based assay for the detection of serum anti-BG-A_tri_ IgM.

## Materials and methods

The general procedures and materials are described below. A full, detailed Standard Operating Procedure (SOP) is included in the Supporting Information (Appendix A in S1 File).

### Serum samples

Anti-BG-A_tri_ IgM values were measured for two subject groups: healthy subjects and prostate cancer patients. Sera from healthy individuals were purchased from Valley Biomedical Products and Services (Winchester, VA) (n = 70) and from Bioreclamation LLC (Westbury, NY) (n = 58). Samples were accompanied by a certification that all samples were tested in accordance with FDA regulations and found to be negative for HIV ½ AB, HCV AB, and non-reactive for HBSAG, HIV-1 RNA, HCV RNA, and STS. All samples were stored at –80°C or –20°C until used. Samples were in storage for approximately 4 years prior to use. Sera from prostate cancer patients on PROSTVAC-VF (n = 45) and controls (n = 32) from a placebo-controlled, multi-center Phase II study of PROSTVAC-VF (NCT00078585) were also evaluated. [3] Across all study centers, sera were obtained in serum separator tubes, processed within 4 hours, and stored at –80°C until assayed. Samples were in storage for approximately 5 years prior to use. All samples had undergone at least one, but not more than 3, freeze-thaw cycles. The study was approved by Institutional Review Board of the National Cancer Institute (NCI). All patients signed a consent form approved by the IRB. The following serum samples were also used as controls in every assay: Negative serum [Liquicheck Rheumatoid Factor Control, Level 1 (BioRad, 501); undetectable anti-BG-A_tri_ IgM], High Positive serum [Human Serum (Male)–BioreclamationIVT—HMSRM-M], and Low Positive [WHO Rheumatoid Factor Reference Serum (NIBSC, Part Number W1066)]. Hemolyzed serum samples were prepared by mixing serum with 1–2% whole blood.

### Luminex assay

Antigens were coupled to Luminex magnetic microspheres (Luminex—MC10013-01, MC10030-01, MC10045-01, MC10073-01) using a Luminex coupling kit (Luminex—40–50016) following the manufacturer’s instructions (see Supporting Information SOP for additional details). The Blood Group A neoglycoprotein (BG-A_tri_—6 atom spacer BSA; V-Labs—NGP6305) was coupled to bead region 13. The Galili neoglycoprotein (Galα1-3Galβ1-4Glc-3 atom spacer BSA; V-Labs—NGP0330) was coupled to bead region 30. Albumin from bovine serum (Sigma—A7888) was coupled to bead region 45, and IgG from human serum (Sigma—I4506) was coupled to bead region 73.

Each serum sample was evaluated in triplicate following the SOP. Briefly, serum samples were diluted 1:50 in PBS buffer and 50 μL was added to the respective wells according to the 96-well plate layout described in the Supporting Information. To each well was added 50 μL of bead mixture (solution containing 50 beads/μL of each bead set in PBS-TBN containing 1% BSA). The plate was incubated in the dark for 1 hour and shaken at 800 rpm. The plate was placed on a magnetic plate separator (Magnetic Plate Separator, Luminex, CN-0269-01, center capture) and the supernatant was removed. The beads were then washed 2 times with 100 μL PBS-TBN (0.1% BSA). Next, 100μL of detector antibody solution (biotinylated AffiniPure Goat Anti-Human IgM, Fc5μ Fragment Specific, Jackson ImmunoResearch– 109-005-043; see Supporting Information) was added to each well and the plate was incubated for 30 min and 800 rpm at room temperature in the dark. The supernatant was removed and the beads were washed twice. Next, the beads were incubated with 100μL/well Streptavidin—Phycoerythrin conjugate (Moss, Inc., SAPE-001; diluted to 4μg/mL in PBS-TBN 1% BSA) for 30 min and shaken at 800 rpm at room temperature in the dark. The supernatant was removed and the beads were washed twice. Next, 100μL of PBS-TBN 0.1% was added to each well, the plate was incubated for 30 sec. and shaken at 800 rpm at room temperature in the dark, and then immediately analyzed using a Luminex 200 analyzer and Luminex xPONENT software version 3.1 Build 971 (Luminex)

### Data processing and analysis

All bead counts were checked and any that were below 35 were excluded from further analysis. External Serum Controls (Negative serum, High Positive serum, and Low Positive serum) were then checked for assay tolerance ranges (see Supporting Information for details), and plates with control values outside the acceptable range were repeated. Next, the IgG beads were evaluated for Rheumatoid factor levels (Rf levels). The sample IgG assay (bead region 73) MFI value was divided by the mean Negative Serum Control MFI value (bead region 73, IgG assay). Samples with 0.75 or greater value were flagged and tested separately to determine their Rf levels (see Supporting Information). The BSA assay MFI signal (bead region 45) was subtracted from the BG-A_tri_ MFI signal (bead region 13) from the same well to get the BG-A_tri_ signal.

Intra-plate variability was calculated by determining the mean and standard deviation of all replicates on a plate. The standard deviation was calculated as $\sqrt{\frac{\sum {\left(x-\bar{x}\right)}^{2}}{\left(n-1\right)}}$ where $\bar{x}$ is the mean and *n* is the number of replicates. The coefficient of variation (%CV, also known as the relative standard deviation) was calculated as the standard deviation divided by the mean value, and expressed as a percent (multiply by 100). To determine inter-plate variability, all replicates across all plates were pooled together to determine the mean and standard deviation. The %CV was calculated as described above using the pooled mean and standard deviation values.

Finally, MFI values were normalized and converted to International Units (IU) for the BG-A_tri_ assay to standardize the results (see Figure D and Tables D and E in S1 File for full details). The BG-A_tri_ value for the WHO sample was used to estimate the IU for BG-A_tri_. The WHO sample had a known value in IU for IgG, but not for BG-A_tri_. We approximated the BG-A_tri_ IU values by using data from the IgG WHO curve from the same three experiments done in triplicate. The MFI value of the reference sample was determined in IU using the WHO results; the reference serum was found to have 2.85 IU (2,185 mIU) of BG-A_tri_ in the sample. Lastly, the IU value of the reference serum was used to calculate the concentration in IU of the standard curve, based on the dilution series. The analytical validation results were then interpolated using these concentrations. The standard curve on each plate was fitted using a five parameter logistic curve (Richard’s model, [13] which is a modified Hill equation) since these curves are asymmetric around the midpoint.

### Comparison of microarray data and Luminex data

To determine if the Luminex assay provides signals that are consistent with the microarray data, several comparisons were made. For each individual sample, the Luminex assay signal was plotted versus the microarray signal. The data was fitted to a linear regression and the Pearson correlation coefficient was determined. In addition, the signals were ranked from highest value to lowest value for the microarray data and the Luminex data. Luminex assay rankings were plotted versus the microarray rankings and the Pearson correlation coefficient was determined. Lastly, to determine if there is bias based on signal strength, the difference in signal between the measured Luminex signal and the expected Luminex signal based on the linear regression (Luminex vs microarray) was plotted as a function of the microarray signal (Figures B and C in S1 File).

## Results

### Development of the assay

**Fig 1:**
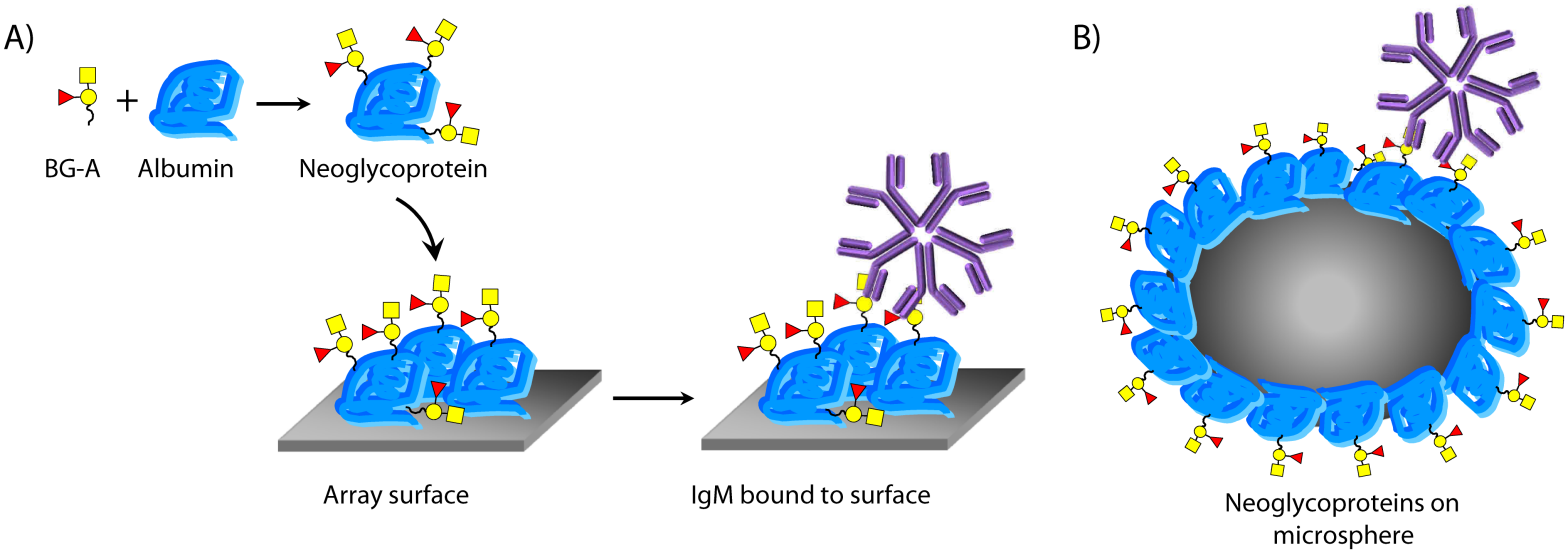
Overview of neoglycoproteins and assay formats. Glycans or glycopeptides are covalently coupled to albumin to produce neoglycoproteins, which are then printed onto a microarray surface and used to detect IgM to BG-A_tri_. Neoglycoproteins immobilized on Luminex microspheres mimic glycan presentation on the microarray surface.

Two key considerations were the assay platform and the antigen format. The glyco-antigen microarray that we used for the initial discovery and validation contained neoglycoproteins printed on an epoxide-coated glass microscope slide (Fig 1). [14–16] A neoglycoprotein is a protein-carbohydrate conjugate containing one or more copies (usually 5–25) of a glycan determinant attached covalently to a carrier protein, typically bovine serum albumin (BSA). [17,18] We hypothesized that by using the same neoglycoproteins in a clinical assay, we would be able to capture the same antibody populations being captured on the array. For the assay platform, we decided to use the Luminex bead-based assay format. This platform is FDA-approved for diagnostic use, has high sensitivity, is versatile, is amenable to screening large numbers of patient samples, and is fully compatible with the use of neoglycoproteins as the capture antigen. [19,20]

We opted to develop a four-plex assay. In addition to measuring anti-BG-A_tri_ IgM levels, we also included three controls: Galili antigen (alpha-Gal antigen; Galα1-3Galβ1-4Glc), BSA, and IgG. Since the vast majority of humans have high antibody levels to the Galili antigen, this antigen serves as a positive control. Unmodified BSA was included as a negative control to detect antibodies that may bind the BSA carrier protein. Finally, some humans have IgM antibodies to IgG, referred to as rheumatoid factors (Rf). If these individuals have IgG that bind BG-A_tri_, then the IgM rheumatoid factors could give rise to a false IgM level for the BG-A_tri_ beads. BG-A_tri_ -BSA conjugate, and three controls (Galili-BSA conjugate, BSA, and IgG) were coupled to a different bead set for the multiplex assay.

During the course of these studies, several technical challenges arose. First, we found considerable variability from one lot of secondary antibody to another. Therefore, we implemented a secondary antibody qualification/assessment step to ensure batches of secondary reagent met our criteria prior to use (see Supporting Information for details). Second, we anticipated potential problems due to variability over time of reagents and/or instrumentation. Moreover, clinical applications would require standardization of anti-BG-A_tri_ IgM levels. Therefore, we developed a standard curve using human male Type B serum pooled from ten healthy individuals. Blood Type B individuals generally have high anti-BG-A_tri_ IgM levels. [12,21] Each measurement for each serum sample was interpolated to the standard curve. Since the amount of anti- BG-A_tri_ (in mg) in our reference serum was unknown, we normalized the signal relative to the WHO reference serum, and then calculated the BG-A_tri_ values for each sample. The WHO sample and the reference serum would be run on every plate and used to convert anti-BG-A_tri_ IgM signals into International Units (IU) to standardize the values.

We initially developed the assay for measuring anti-BG-A_tri_ IgM in singleplex. A wide range of serum dilutions were evaluated to identify conditions providing significant signal above the background as well as signals in the linear range; these data were used to develop the standard curve for the assay. We evaluated four serum samples containing a range of high, medium, medium-low, and low IgM to BG-A_tri_ and determined that dilutions in the range of 1:50 to 1:100 were acceptable for samples ranging from low to high anti-BG-A_tri_ IgM, with %CV under 6% (Table 1).

**Table 1:** Selection of sample dilution.^a^

%CV	Low	MedLo	Med	High
ALL	32.12%	24.73%	6.72%	13.14%
**1:50, 1:100**	**5.88%**	**4.68%**	**4.76%**	**4.44%**
1:25, 1:50, 1:100	8.05%	6.08%	5.67%	17.41%
1:50, 1:100, 1:200	16.39%	10.50%	5.08%	5.24%
1:100, 1:200, 1:400	26.46%	21.63%	2.28%	4.31%

^**a**^ Four samples with previously characterized anti-BG-A_tri_ IgM levels of low, medium-low, medium and high values were tested at five different dilutions (1:25, 1:50, 1:100, 1:200 and 1:400). Each result was interpolated to the standard curve, adjusted for dilution, and %CVs were calculated in comparison to all four dilutions or a subset. Two dilutions at 1:50 and 1:100, provided good agreement (after adjusting for dilution factor) across all four samples, with %CV under 6% for all samples.

Next, we evaluated specificity to detect anti-BG-A_tri_ IgM and potential cross-reactivity. To confirm that the signals were specific and not due to nonspecific interactions, we pre-incubated serum with BG-A_tri_ neoglycoprotein as a competitive inhibitor and demonstrated a substantial, dose-dependent decrease in signal (Table 2). At the highest dilution tested (1:1,000), the signal to background went from almost 200 to around 5 in the presence of soluble BG-A_tri_ neoglycoprotein. Serum was also depleted of either BG-A antibodies or Galili antibodies prior to evaluation in singleplex versus each of the 4 antigens using beads with conjugated BG-A_tri_ neoglycoprotein. No cross reactivity was observed (data not shown).

**Table 2:** Assay specificity^a^.

Serum dilution	Signal:Background		Fold change
	−BG-A	+BG-A	
1:20,000	5.1	1.5	3.3
1:10,000	18.0	1.2	15.1
1:5,000	39.1	1.5	25.5
1:4,000	47.7	2.4	20.3
1:1,000	197.7	4.6	42.6

^**a**^ Assay specificity. When positive serum was pre-incubated with 25mg/mL BG-A, a substantial, dose-dependent decrease in signal is observed.

**Fig 2:**
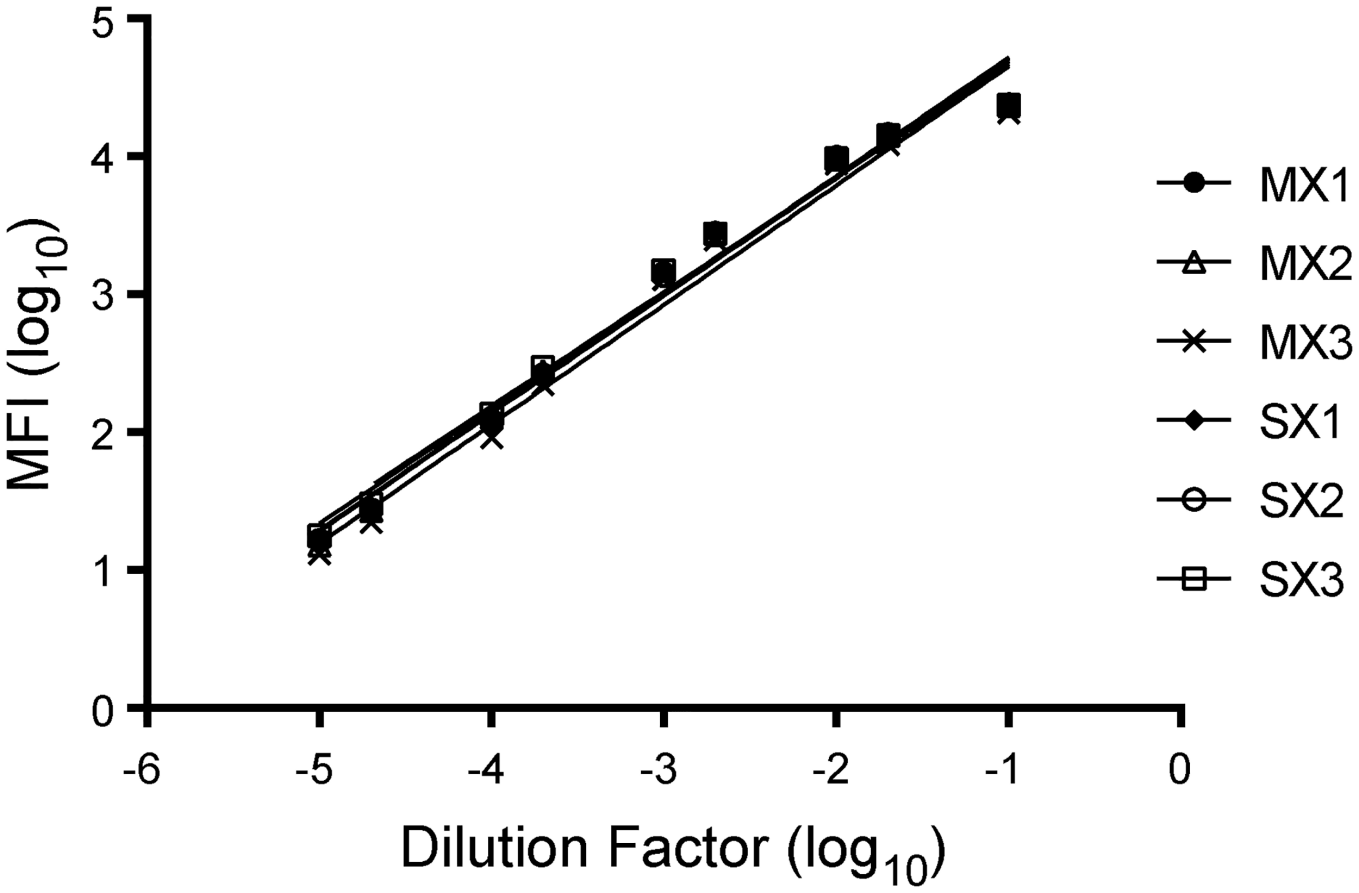
Comparison of singleplex and multiplex measurements of IgM to BG-A_tri_. BG-A_tri_ signals were measured in triplicate across a range of serum concentrations (MX—multiplex; SX—singleplex).

Next, we evaluated potential complications due to multiplexing. To determine the effects of multiplexing on the assay and to verify the standard curve, a pooled serum sample was assayed across a broad dilution range in triplicate over 3 days in a singleplex or multiplex format. Singleplex data for each of the BG-A_tri_ antigen and controls were compared with the multiplex data. Signals were nearly identical for singleplex and multiplex assays for BG- A_tri_ as well as the three controls (Fig 2 and Figure A in S1 File), demonstrating that there was no intra-assay interference. Assays for BG-A_tri_ and Galili antigens had %CVs below 10%, whereas the BSA and IgG assays had %CVs below 20% (data not shown). For the BG-A_tri_ assay, signals correlated linearly with dilution in the range of 1:50 to 1:100,000; however, a dilution of 1:10 produced signals that were close to saturation.

### Analytical validation of the assay

We evaluated potential complications due to sample stability, interference, and carryover from well-to-well. Signal reproducibility was evaluated after three serum samples (reference serum, high and low) were thawed and refrozen three times, and no significant change was observed for any of the four antigens, with %CV generally under or near 10% (data not shown). Low, medium and high serum samples were also subjected to hemolysis and addition of bilirubin to determine if the presence of either would interfere with the assay. Addition of bilirubin had no significant effect on any of the signals, with %CV generally less than 10%. Hemolysis did not affect the high and low samples, but the medium sample demonstrated a decrease in BG-A_tri_ signal of 20% and Galili of 40%. Thus, hemolysis may affect anti-BG-A_tri_ IgM signals for some samples. We also evaluated potential carry-over or cross-contamination from well-to-well on the 96-well plates, but none was detectable (see Supporting Information for details).

Reproducibility is a critical feature for a clinical assay. CVs can vary considerably based on signal strength, with low signals often having higher variability than medium or high signals. In addition, reproducibility of signals from human serum can vary from one person to another due to variations in serum compositions. To evaluate reproducibility, we assessed 6 different serum samples: two with high anti-BG-A_tri_ IgM, two with medium signals, and two with low signals. Five of these samples were known to be positive for anti-BG-A_tri_; the sixth sample (Sample E) was negative. Each sample was assayed at two dilutions: 1:50 and 1:100. Each sample/dilution combination was tested a total of 96 times, with 12 replicates per plate across 8 plates. Two different analysts performed the assays, with half the samples carried out by each. For the entire set of 16 plates, there were only 5 wells (<0.5%) that had too few beads to collect data.

We assessed reproducibility for both the raw measured values and the standardized values determined in IU. For the raw values, the assay produced highly reproducible results with all intra-plate %CVs for BG-A_tri_ below 10% and all inter-plate %CVs below 13% (see Supporting Information, Tables A and B in S1 File). We also evaluated reproducibility after normalizing and converting to IU. Most samples had %CVs that were ≤14% (Table 3, and Table C in S1 File). The sample with very low anti-BG-A_tri_ IgM and the sample with the highest anti-BG-A_tri_ IgM had higher variability; however, these samples would be considered anti-BG-A_tri_ negative and anti-BG-A_tri_ positive, respectively, at all measured values. Furthermore, Sample B at 1:100 dilution (6,152.1 mIU) and Sample F at 1:100 dilution (53.5 mIU) (Table 3) were the highest and lowest positive samples tested within the dynamic range of the assay, respectively, and therefore represent the validated Upper Limit of Detection (ULOD) and Lower Limit of Detection (LLOD) of the assay. Taken together, the results demonstrate that reproducibility for the assay is within acceptable limits.

**Table 3:** Analytical validation summary. For the analytical validation, for each sample dilution, we ran eight plates with twelve replicates on each plate (n = 96).

Sample	Anti-BG_Atri_ designation	DF	mIU	Precision (%CV)		Notes
				Intraplate	Interplate	
			n = 96	n = 12*	n = 96	
A	Positive	1:100	2,198.5	4.6%	8.5%	
		1:50	2,072.9	6.4%	11.2%	
B	Positive	1:100	6,152.1	7.0%	11.8%	validated assay ULOD
		1:50	7,687.2	32.4%	54.8%	above assay dynamic range
C	Positive	1:100	444.3	1.6%	10.7%	
		1:50	452.0	3.5%	10.4%	
D	Positive	1:100	591.2	1.8%	4.7%	
		1:50	560.1	3.3%	7.3%	
E	Negative	1:100	16.9	3.6%	35.0%	below LLOD
		1:50	12.3	2.9%	20.7%	below LLOD
F	Positive	1:100	53.5	3.8%	10.8%	validated assay LLOD
		1:50	54.5	2.6%	14.2%	

*value shown is the mean %CV of the intraplate precision from all eight plates.

In addition to evaluating reproducibility, we also established tolerance ranges for the assay. Each of the serum control samples was assayed on 5 plates on 5 separate days. The mean value and standard deviations were determined for BG-A_tr_ and Galili antigen positive control, and the tolerance ranges were set as plus or minus 3 standard deviations (Table 4). Values falling out of this range would indicate a technical problem with the plate and all data on that plate would be retested.

**Table 4:** Tolerance ranges for the assay.

Antigen	Serum Sample	Mean^a^	SD	Min	Max
BG-A_tri_	Neg Serum 1:50	12.6	11.0	0	47
BG-A_tri_	High Pos Serum 1:50	2,799	418	1,545	4,053
BG-A_tri_	Low Pos Serum 1:50	40.8	6.6	20	61
Galili	Neg Serum 1:50	46.3	6.1	28	65
Galili	High Pos Serum 1:50	3,127	392	1,951	4,303
Galili	Low Pos Serum 1:50	59.5	7.1	38	81

^a^Tolerance ranges were defined by the mean (mIU) plus/minus 3 standard deviations

### Cross-platform comparison

**Fig 3:**
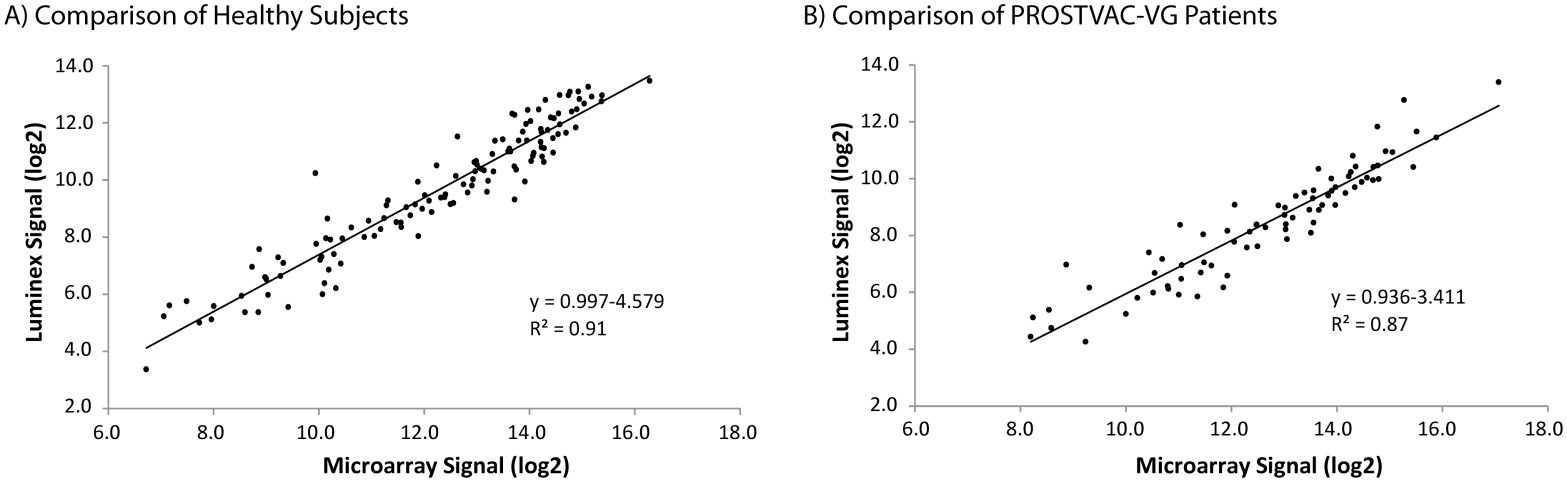
Comparison of BG-A_tri_ signals measured using the Luminex assay and the microarray assay.

The final assessment of the assay involved evaluating the consistency of signals measured with the Luminex assay and the glyco-antigen microarray assay. The cross-platform comparison involved two stages. In the first stage, we compared anti-BG-A_tri_ IgM from the Luminex assay with signals measured using the glyco-antigen microarray for 126 healthy subjects. The array signals were measured previously at a dilution of 1:50. [12] For the Luminex assay, samples were evaluated at 1:50 and 1:200 in triplicate. For each individual sample, the Luminex assay signal was plotted versus the microarray signal (Fig 3A). Using this approach, the data for the two assays was found to be linearly correlated with an R value of 0.95 (Pearson correlation). In addition, the Spearman correlation based on rankings was also high, with an R value of 0.95. It is important to note that the data were evenly distributed about the trend line from low to high values, indicating no systematic bias at high or low signal strength (see Figure B in S1 File).

Next, we compared signals from the two assay platforms for 77 prostate cancer patients from a Phase II clinical trial of PROSTVAC-VF. The microarray signals were measured previously at a dilution of 1:50. [11,14] These samples were then evaluated using the Luminex assay at a dilution of 1:50 as well. A scatterplot of microarray data versus Luminex data was then generated (Fig 3B). As with the healthy samples, these samples demonstrated highly correlated results between the two assays, with an R value of 0.93 for the signals and 0.95 for the Spearman correlation based on rankings. The data were evenly distributed about the trend line from low to high values, indicating no systematic bias based on signal strength (see Figure C in S1 File).

## Discussion

In a retrospective study, we previously found that anti-BG-A_tri_ IgM levels measured prior to treatment correlated positively with overall survival of cancer patients on PROSTVAC-VF therapy from two different clinical trials. [11] That study suggested that measurement of anti-BG-A_tri_ IgM from patient serum prior to treatment could provide a simple approach to identify patients that are likely to benefit from PROSTVAC-VF. Identifying patients that are likely to benefit from a given therapy could significantly improve patient care by maximizing clinical benefits while minimizing adverse effects.

The initial discovery and preliminary validation were based on a glyco-antigen microarray assay. The array contained hundreds of oligosaccharides, polysaccharides, and glycopeptides immobilized on a solid support in a spatially defined arrangement. While the microarray format is a powerful tool for biomarker discovery, it is not an ideal format for clinical assays. To translate our discovery into a clinically useful biomarker, we needed a more economical and clinically viable assay format. While glyco-antigen arrays have been used to identify other candidate biomarkers, [14,16,22–33] successful conversion from a microarray-based research grade assay to a clinical assay has not been reported.

Conversion of a research grade assay into a clinical assay presents numerous challenges. [34,35] Research assays are typically designed for initial discovery and often use specialized equipment, lab specific expertise, and expensive reagents and supplies. Clinical assays, however, must meet rigorous performance standards, be resistant to operator variability, provide reproducible results that are consistent over long periods of time, and be suitable for evaluating thousands of patients in a cost-effective manner. Migration of our glyco-antigen microarray assay to a clinical assay presented additional challenges. Glycan presentation is a critical feature for capturing and detecting carbohydrate-binding antibodies. [11,36–39] Most carbohydrate-antibody interactions rely on multivalent complex formation to achieve tight binding. Therefore, the spacing and orientation of the carbohydrates must be appropriate to interact with multiple binding sites on the IgM. Since different antibody clones can bind BG-A in different ways (e.g. end-on vs groove binding mode, deep vs shallow pocket, recognition from different angles), the optimal spacing and orientation can vary from one antibody to another, even when they are of the same isotype. [38] Consequently, factors such as glycan density, linker length, and linker flexibility can have a major impact on which antibodies within the polyclonal sera bind to a surface. Our goal was to capture the same population of antibodies that was captured on the microarray. Therefore, we needed a clinical assay format with similar glycan presentation as the microarray surface.

The glyco-antigen microarray that we used contains neoglycoproteins printed on an epoxide-coated glass microscope slide (Fig 1). [14–16] We anticipated that by using the same neoglycoproteins in a clinical assay, many aspects of spacing and orientation on the new surface would be similar to the array surface (Fig 1). For example, the linker flexibility and composition, carrier protein, and valency would be identical in the new assay. While the use of neoglycoproteins was predicted to facilitate migration of the microarray assay to a clinical assay, we had never tested this hypothesis in a rigorous manner. Therefore, key objectives of this study were to determine how readily our approach would translate to a clinical assay and to provide a quantitative comparison of the microarray assay and clinical assay. We note that presentation of the terminal structure of blood group A antigen as a neoglycoprotein may be different than is found on a cell surface or a poxvirus; however, our goal was to capture the population of antibodies with the same specificity that were captured in the original array experiments since that is the key population that correlates with survival.

For this clinical assay platform, we selected the Luminex bead-based assay format. The Luminex platform uses fluorescently encoded microspheres for capturing and detecting target molecules. [19] Each antigen or control is attached to a unique bead set, and then multiple bead sets are mixed together allowing detection of multiple serum antibody subpopulations in a single assay. This platform is FDA-approved for diagnostic use, has high sensitivity, is versatile, and is amenable to screening large numbers of patient samples. [19,20] In addition, the Luminex system is fully compatible with the use of neoglycoproteins as the capture antigen.

Using the neoglycoproteins and the Luminex bead based assay, we successfully developed and validated a clinical assay for monitoring anti-BG-A_tri_ IgM antibody levels in the serum of prostate cancer patients. BG-A_tri_ neoglycoprotein and several controls were immobilized on magnetic beads and used as IgM capture agents. Through a series of optimization steps, a well-defined standard operating procedure (SOP) was established leading to an assay that meets rigorous clinical performance standards. Methods are in place to standardize signals across laboratories, and the assay has undergone extensive analytical validation to ensure reproducible results. We demonstrated that the Luminex-based assay and the microarray assay produce highly correlated results in two separate groups, 126 healthy subjects and 77 PROSTVAC-VF treated patients. The high correlations indicate that the two assays are capturing the same, or very similar, antibody subpopulations in serum. The assay is a Laboratory Developed Test (LDT) and is suitable for evaluating thousands of serum samples in CLIA certified laboratories that have validated the assay. Thus, we now have an assay that is suitable for evaluating serum samples from the approximately 1200 patients in the Phase III clinical trial. Taken together, the results demonstrate successful migration of a laboratory grade microarray assay to a clinical assay and highlight the utility of neoglycoproteins for glyco-antigen microarrays.

## Supporting information

S1 File Supporting experiments, tables, and figures. (PDF)
