# Supplementary material for: Development and validation of a Luminex assay for detection of a predictive biomarker for PROSTVAC-VF therapy
Source: PLoS One. 2017 Aug 3;12(8):e0182739. doi: 10.1371/journal.pone.0182739 (PMC5542629; doi:10.1371/journal.pone.0182739)
Supplement: S1 File — (PDF) [file pone.0182739.s001.pdf]

Supporting Information For

Development and Validation of a Luminex Assay for Detection of a Predictive Biomarker for PROSTVAC-VF Therapy

Julie L. Lucas,[1] Erin A. Tacheny,[1] Allison Ferris,[1] Michelle Galusha,[1] Apurva K. Srivastava[2], Aniruddha Ganguly,[3] P. Mickey Williams,[4] Michael C. Sachs,[5,#a] Magdalena Thurin,[3] James V. Tricoli,[6] Winnie Ricker,[7] and Jeffrey C. Gildersleeve[8]*

Affiliations: [1]MRIGlobal, 65 West Watkins Mill Rd, Gaithersburg, MD; [2]Pharmacodynamics Biomarker Program, Applied/Developmental Research Directorate Frederick National Laboratory for Cancer Research, Leidos Biomedical Research, Inc., Frederick, MD; [3]Cancer Diagnosis Program, Division of Cancer Treatment and Diagnosis, National Cancer Institute, National Institutes of Health, Bethesda, MD; [4]Molecular Characterization and Clinical Assay Development Laboratory, Frederick National Laboratory for Cancer Research, Frederick, MD, United States; [5] Biostatistics Branch, Biometric Research Program, NCI, NIH, Bethesda, MD; [6] Diagnostic Biomarkers and Technology Branch, Cancer Diagnosis Program Division of Cancer Treatment and Diagnosis, National Cancer Institute, 9609 Medical Center Drive., Rockville, MD 20892; [7]Information Management Services, Inc., Rockville, MD;

[8]Chemical Biology Laboratory, Center for Cancer Research, National Cancer Institute, National Institutes of Health, Frederick, MD

[#a] current address: Biostatistics Unit, Institute of Environmental Medicine, Karolinska Institutet, Stockholm, Sweden;

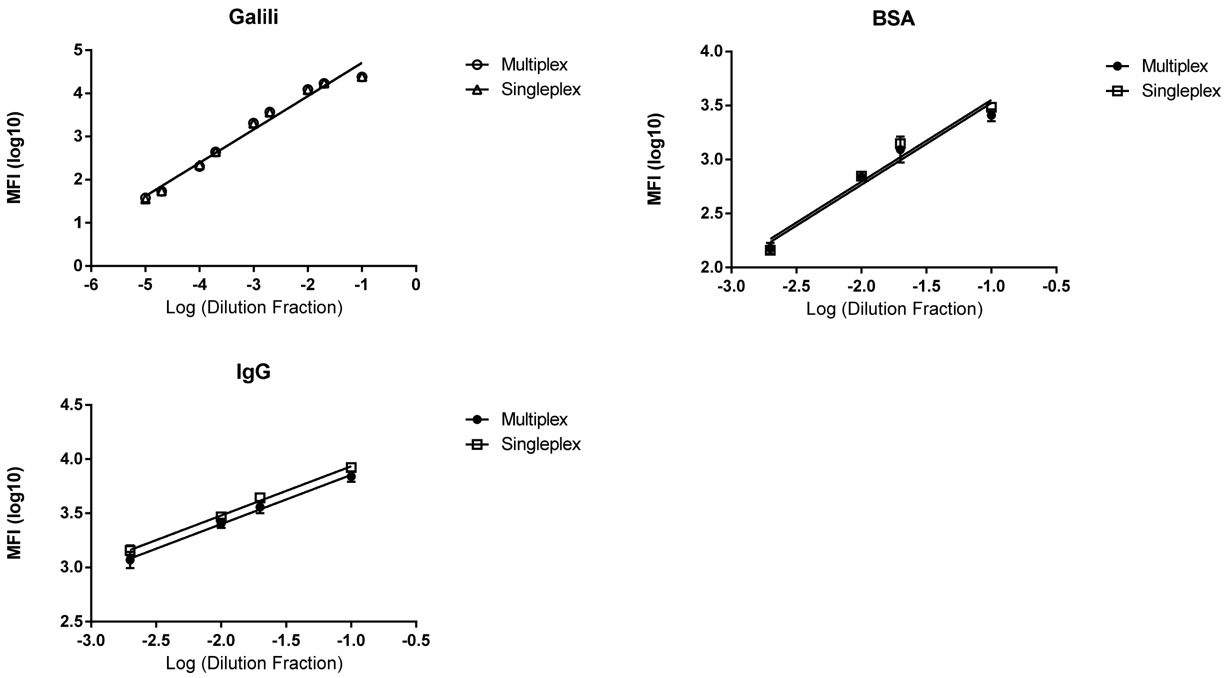

Figure A. Evaluation of singleplex vs multiplex data: A pooled serum sample was assayed across a broad dilution range in triplicate over 3 days for each of the 4 antigens in a singleplex format and then also in a multiplex format. Data for BG-Atri is shown in the main paper. Data for Galili, BSA, and IgG are shown above.

Evaluation of potential carry over and cross-well contamination: Samples with very low BG-A_{tri} levels (E and F in the grid below) were diluted 1:50 or 1:100 and then positioned either in the upper left corner of the plate or intermixed among very high BG-A_{tri} samples (e.g. A at 1:50; see below). BG-A_{tri} levels were then evaluated and compared between samples in the two regions to detect potential carry over or cross-well contamination. Positioning on the plate had no significant effect on measured signals. In addition, the negative control samples in the upper left and the one in the lower center right (surrounded by high signal wells) were all negative in the assay indicating no contamination. The assay was repeated on 6 different days.

Plate 1A: simplified (only run A, C, and E)

	1	2	3	4	5	6	7	8	9	10	11	12
A	Negative Control	Negative Control	E 1:100	E 1:50	C 1:100	C 1:100	C 1:100	C 1:50	A 1:50	A 1:100	A 1:100	C 1:50
B	E 1:100	E 1:100	E 1:100	E 1:50	C 1:100	C 1:100	C 1:100	A 1:50	E 1:100	A 1:50	A 1:100	C 1:50
C	E 1:100	E 1:100	E 1:50	E 1:50	C 1:100	C 1:100	C 1:50	C 1:50	A 1:50	C 1:50	A 1:100	C 1:50
D	E 1:50	E 1:50	E 1:50	E 1:50	C 1:100	C 1:100	C 1:50	A 1:50	E 1:100	A 1:50	E 1:100	A 1:100
E	E 1:50	E 1:50	E 1:50	E 1:50	C 1:100	C 1:100	A 1:100	E 1:100	A 1:50	C 1:50	A 1:50	A 1:100
F	Reference Serum 1:10935	Reference Serum 1:3645	Reference Serum 1:1215	Reference Serum 1:405	Reference Serum 1:135	Reference Serum 1:45	Reference Serum 1:15	A 1:100	C 1:50	A 1:50	E 1:100	A 1:50
G	Reference Serum 1:10935	Reference Serum 1:3645	Reference Serum 1:1215	Reference Serum 1:405	Reference Serum 1:135	Reference Serum 1:45	Reference Serum 1:15	Negative Control	A 1:50	E 1:100	A 1:50	C 1:50
H	Reference Serum 1:10935	Reference Serum 1:3645	Reference Serum 1:1215	Reference Serum 1:405	Reference Serum 1:135	Reference Serum 1:45	Reference Serum 1:15	A 1:100	A 1:100	A 1:100	A 1:100	C 1:50

Plate 1B: simplified (only run B, D, and F)

	1	2	3	4	5	6	7	8	9	10	11	12
A	Negative Control	Negative Control	F 1:100	F 1:50	D 1:100	D 1:100	D 1:100	D 1:50	B 1:50	B 1:100	B 1:100	D 1:50
B	F 1:100	F 1:100	F 1:100	F 1:50	D 1:100	D 1:100	D 1:100	B 1:50	F 1:100	B 1:50	B 1:100	D 1:50
C	F 1:100	F 1:100	F 1:50	F 1:50	D 1:100	D 1:100	D 1:50	D 1:50	B 1:50	D 1:50	B 1:100	D 1:50
D	F 1:50	F 1:50	F 1:50	F 1:50	D 1:100	D 1:100	D 1:50	B 1:50	F 1:100	B 1:50	F 1:100	B 1:100
E	F 1:50	F 1:50	F 1:50	F 1:50	D 1:100	D 1:100	B 1:100	F 1:100	B 1:50	D 1:50	B 1:50	B 1:100
F	Reference Serum 1:10935	Reference Serum 1:3645	Reference Serum 1:1215	Reference Serum 1:405	Reference Serum 1:135	Reference Serum 1:45	Reference Serum 1:15	B 1:100	D 1:50	B 1:50	F 1:100	B 1:50
G	Reference Serum 1:10935	Reference Serum 1:3645	Reference Serum 1:1215	Reference Serum 1:405	Reference Serum 1:135	Reference Serum 1:45	Reference Serum 1:15	Negative Control	B 1:50	F 1:100	B 1:50	D 1:50
H	Reference Serum 1:10935	Reference Serum 1:3645	Reference Serum 1:1215	Reference Serum 1:405	Reference Serum 1:135	Reference Serum 1:45	Reference Serum 1:15	B 1:100	B 1:100	B 1:100	B 1:100	D 1:50

Table A. Mean MFI and % CV for All Samples (1:100).

Assay	Sample Type	Name	Mean MFI	% CV Intra-plate 1:100 Day 1	Day 2	Day 3	Day 4	Day 5	Day 6	Day 7	Day 8	Inter-plate 1:100 All
BG-A_{tri}	High	Sample A	8286	1.3%	1.0%	6.6%	3.0%	5.8%	3.6%	2.6%	3.2%	10.5%
	Medium	Sample C	2137	1.8%	2.1%	1.6%	1.7%	1.3%	1.0%	1.5%	1.0%	10.6%
	Low	Sample E	51	8.8%	2.1%	8.1%	6.9%	4.4%	8.8%	6.2%	4.5%	11.2%
	High	Sample B	15115	1.3%	2.4%	3.0%	5.9%	2.1%	3.1%	2.6%	6.2%	9.6%
	Medium	Sample D	2725	1.8%	1.3%	1.3%	2.6%	1.2%	1.5%	1.4%	1.6%	10.5%
	Low	Sample F	230	4.5%	5.4%	4.8%	3.2%	6.3%	3.3%	4.3%	3.6%	12.7%
Galili	High	Sample A	7270	1.5%	1.3%	5.5%	1.8%	4.7%	2.5%	2.2%	2.8%	12.3%
	Medium	Sample C	18603	2.4%	1.1%	2.3%	2.1%	1.7%	1.2%	2.3%	1.7%	5.8%
	Low	Sample E	7214	1.7%	3.1%	1.7%	2.2%	1.3%	3.0%	1.3%	1.9%	9.5%
	High	Sample B	21046	1.3%	1.9%	2.7%	5.3%	2.0%	3.6%	3.6%	7.0%	6.0%
	Medium	Sample D	9108	1.6%	1.2%	1.2%	3.4%	1.1%	1.1%	1.1%	2.0%	7.8%
	Low	Sample F	2665	2.1%	2.1%	1.5%	1.7%	1.8%	1.1%	1.6%	2.0%	9.6%
BSA	High	Sample A	46	4.6%	4.5%	5.7%	4.3%	5.0%	5.0%	2.1%	2.5%	21.4%
	Medium	Sample C	125	4.9%	2.7%	3.1%	3.0%	2.8%	3.9%	2.1%	3.6%	16.5%
	Low	Sample E	46	7.4%	3.6%	8.1%	4.7%	3.6%	7.9%	6.1%	4.7%	20.2%
	High	Sample B	87	4.2%	5.5%	3.2%	4.9%	4.8%	10.8%	4.0%	7.5%	19.4%
	Medium	Sample D	22	6.3%	4.5%	4.4%	5.5%	4.8%	4.5%	6.8%	4.0%	20.8%
	Low	Sample F	15	8.7%	4.7%	9.2%	7.9%	7.1%	7.9%	8.7%	5.7%	16.9%
IgG	High	Sample A	5368	1.9%	1.1%	8.3%	2.5%	7.0%	4.8%	3.5%	5.1%	11.6%
	Medium	Sample C	2121	2.7%	2.3%	1.8%	1.1%	0.9%	1.1%	2.6%	1.0%	16.7%
	Low	Sample E	8284	4.2%	1.3%	3.5%	2.6%	1.4%	3.6%	2.4%	1.5%	9.0%
	High	Sample B	798	3.4%	3.0%	4.4%	5.5%	1.7%	6.2%	4.1%	5.2%	15.8%
	Medium	Sample D	3394	1.4%	1.0%	1.7%	4.0%	1.3%	1.7%	1.7%	2.0%	9.5%
	Low	Sample F	1467	4.4%	3.8%	4.0%	4.0%	4.0%	3.6%	3.6%	2.7%	9.7%

Table B. Mean MFI and % CV for All Samples (1:50).

Assay	Sample Type	Name	Mean MFI	% CV Intra-plate 1:50 Day 1	Day 2	Day 3	Day 4	Day 5	Day 6	Day 7	Day 8	Inter-plate 1:50 All
$BG\text{-}A_{tri}$	High	Sample A	12568	4.1%	1.1%	5.5%	2.1%	7.0%	3.6%	4.4%	3.3%	10.6%
	Medium	Sample C	4039	4.0%	2.3%	3.4%	3.7%	5.1%	2.1%	1.6%	1.8%	9.3%
	Low	Sample E	94	6.8%	4.7%	5.2%	2.6%	2.2%	4.0%	3.5%	3.6%	12.0%
	High	Sample B	19783	2.2%	2.6%	4.2%	7.1%	3.7%	4.1%	5.2%	5.3%	8.9%
	Medium	Sample D	4751	2.0%	3.4%	3.7%	3.6%	1.3%	2.5%	1.5%	3.9%	8.5%
	Low	Sample F	514	1.8%	3.7%	2.7%	2.4%	2.6%	2.9%	2.7%	3.9%	11.1%
Galili	High	Sample A	11344	3.9%	1.1%	6.0%	2.3%	6.6%	2.4%	4.3%	4.0%	11.3%
	Medium	Sample C	23003	2.5%	0.6%	3.6%	1.2%	6.0%	1.6%	0.8%	3.1%	4.4%
	Low	Sample E	11989	2.8%	1.3%	2.4%	3.6%	3.3%	2.0%	1.8%	1.4%	7.8%
	High	Sample B	23836	0.6%	1.5%	0.8%	5.9%	1.5%	0.9%	2.6%	1.6%	4.6%
	Medium	Sample D	13485	1.4%	2.7%	2.2%	3.5%	1.7%	2.5%	1.4%	3.9%	6.4%
	Low	Sample F	4751	1.1%	1.7%	1.0%	2.0%	1.3%	1.3%	1.1%	2.6%	8.9%
BSA	High	Sample A	73	6.9%	6.1%	12.5%	5.8%	11.3%	7.9%	7.2%	6.0%	17.8%
	Medium	Sample C	248	9.7%	4.2%	9.5%	5.8%	6.1%	8.3%	5.6%	2.3%	13.0%
	Low	Sample E	91	6.0%	4.0%	6.1%	4.0%	2.8%	3.8%	3.5%	3.5%	28.2%
	High	Sample B	146	10.6%	10.6%	3.5%	8.2%	12.9%	14.3%	7.2%	17.4%	15.9%
	Medium	Sample D	30	5.3%	8.5%	5.1%	4.8%	4.7%	4.7%	6.8%	4.0%	24.5%
	Low	Sample F	24	5.4%	6.8%	4.9%	5.0%	3.3%	4.6%	6.0%	4.3%	15.5%
IgG	High	Sample A	7953	3.0%	1.3%	5.3%	3.0%	7.5%	2.2%	4.0%	4.1%	13.3%
	Medium	Sample C	3447	5.1%	1.8%	5.0%	3.4%	5.4%	1.9%	2.3%	3.1%	13.2%
	Low	Sample E	12041	4.1%	1.2%	3.8%	3.0%	2.5%	2.4%	1.3%	1.5%	8.1%
	High	Sample B	1369	6.0%	8.4%	3.1%	6.9%	5.7%	8.3%	4.0%	17.5%	16.6%
	Medium	Sample D	5103	2.9%	3.9%	2.0%	4.2%	2.0%	2.6%	2.3%	6.2%	8.3%
	Low	Sample F	2817	1.3%	1.7%	1.8%	2.7%	2.2%	2.1%	1.7%	2.6%	8.0%

Table C. Mean Interpolated Values in mIU for All Samples – BG-A Assay. MFI values were interpolated and converted to mIU . Sample B 1:50 was outside the linear range of the assay; 1:100 results are more accurate for this sample.

Interpolated (mIU)	High	High	Medium	Medium	Low	Low	High	High	Medium	Medium	Low	Low
	Sample A 1:50	Sample A 1:100	Sample C 1:50	Sample C 1:100	Sample E 1:50	Sample E 1:100	Sample B 1:50	Sample B 1:100	Sample D 1:50	Sample D 1:100	Sample F 1:50	Sample F 1:100
Day 1 Avg	2,175.8	2,329.3	453.0	453.3	11.9	16.6	6,997.4	6,525.6	501.1	544.4	46.5	46.2
**%CV**	**7.3%**	**1.9%**	**4.7%**	**1.9%**	**4.8%**	**5.0%**	**8.3%**	**3.0%**	**2.3%**	**1.9%**	**1.6%**	**3.6%**
Day 2 Avg	2,100.3	2,118.0	409.2	402.6	8.9	10.6	6,921.5	6,098.1	509.0	572.3	53.1	49.9
**%CV**	**2.1%**	**1.4%**	**2.5%**	**2.1%**	**4.2%**	**1.9%**	**11.4%**	**6.0%**	**4.0%**	**1.5%**	**3.6%**	**5.1%**
Day 3 Avg	2,218.7	2,189.0	499.1	467.2	17.0	28.1	5,687.8	5,315.5	554.7	577.6	66.2	61.8
**%CV**	**8.4%**	**9.4%**	**4.5%**	**2.1%**	**1.9%**	**1.7%**	**11.3%**	**5.8%**	**4.3%**	**1.4%**	**2.5%**	**4.5%**
Day 4 Avg	2,117.5	2,266.3	512.3	500.2	14.3	21.2	8,767.1	7,083.6	617.9	628.2	52.9	54.6
**%CV**	**3.3%**	**3.8%**	**4.2%**	**1.9%**	**1.5%**	**2.9%**	**61.4%**	**14.5%**	**4.3%**	**3.0%**	**2.1%**	**2.2%**
Day 5 Avg	1,888.3	2,225.1	490.5	512.5	11.1	10.9	6,501.4	6,369.4	580.8	607.6	67.9	61.8
**%CV**	**11.8%**	**7.7%**	**5.6%**	**1.3%**	**2.5%**	**6.2%**	**10.0%**	**4.3%**	**1.6%**	**1.3%**	**2.4%**	**6.5%**
Day 6 Avg	1,666.0	1,845.3	388.8	391.2	11.0	15.7	6,754.3	6,420.1	562.8	595.1	50.3	51.7
**%CV**	**5.3%**	**4.6%**	**2.5%**	**1.1%**	**2.5%**	**3.7%**	**14.3%**	**6.3%**	**3.0%**	**1.6%**	**2.7%**	**2.5%**
Day 7 Avg	2,202.0	2,342.1	454.3	451.6	14.5	21.0	11,749.7	5,879.3	571.3	584.4	48.1	49.6
**%CV**	**7.4%**	**3.6%**	**1.9%**	**1.6%**	**2.1%**	**2.7%**	**48.5%**	**5.3%**	**1.8%**	**1.6%**	**2.5%**	**3.1%**
Day 8 Avg	2,214.7	2,272.8	408.6	376.2	10.0	10.9	8,727.0	5,524.7	583.3	620.3	50.6	52.6
**%CV**	**5.6%**	**4.3%**	**2.1%**	**1.0%**	**3.8%**	**5.0%**	**93.6%**	**11.0%**	**4.7%**	**1.8%**	**3.6%**	**2.6%**
ALL Avg	2,072.9	2,198.5	452.0	444.3	12.3	16.9	7,687.2	6,152.1	560.1	591.2	54.5	53.5
**%CV**	**11.2%**	**8.5%**	**10.4%**	**10.7%**	**20.7%**	**35.0%**	**54.8%**	**11.8%**	**7.3%**	**4.7%**	**14.2%**	**10.8%**

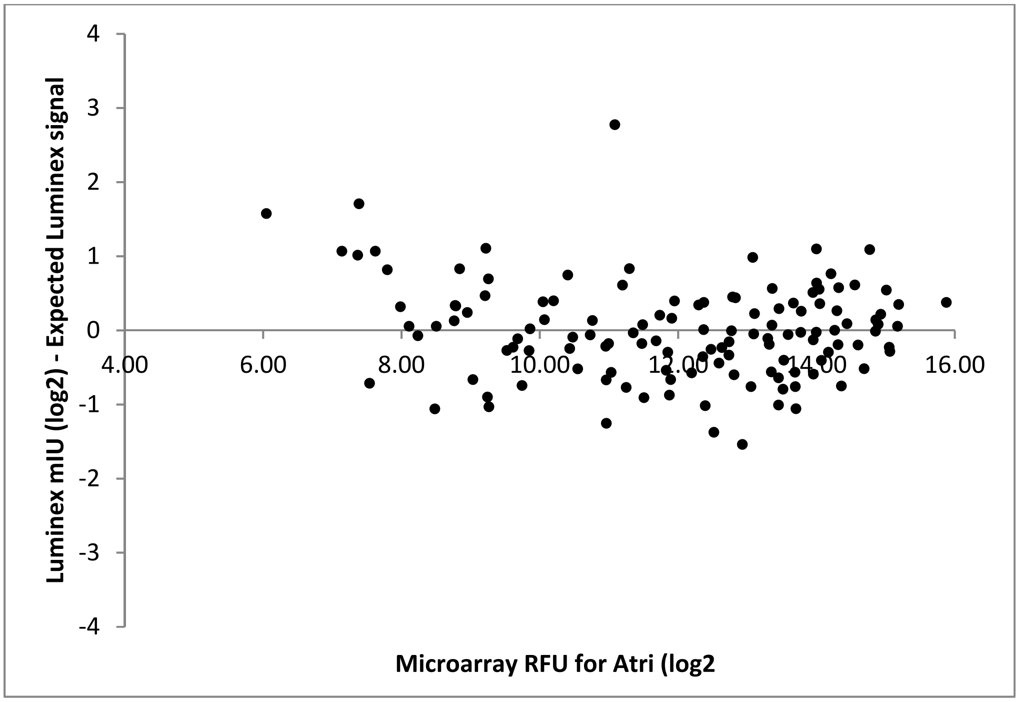

Figure B. Variation in Signals as a Function of Signal Magnitude for Healthy Subjects. The difference in signal between the measured Luminex signal and the expected Luminex signal based on the linear regression (Luminex vs microarray) was plotted as a function of the microarray signal. The data demonstrate no significant bias in variation as a function of signal.

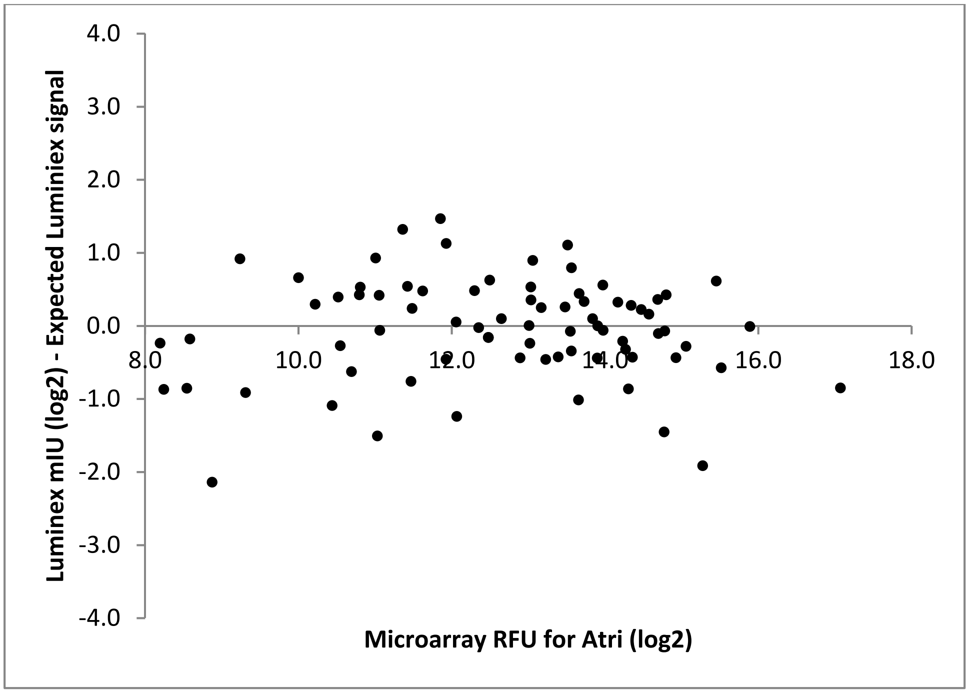

Figure C. Variation in Signals as a Function of Signal Magnitude for PROSTVAC Patients. The difference in signal between the measured Luminex signal and the expected Luminex signal based on the linear regression (Luminex vs microarray) was plotted as a function of the microarray signal. The data demonstrate no significant bias in variation as a function of signal.

Conversion of MFI to IU

To approximate the IU of the reference serum, a serial dilution of the WHO sample was run in triplicate over three separate experiments. In parallel, a 1:100 dilution of the reference serum was also run (see Table S4, below); this dilution is within the linear range of the assay. The results of the IgG assay were fitted to a curve (see Figure S3), and the MFI values of the reference serum (BG-A_{tri} assay) were interpolated to this curve to get approximate IU (Table S5). The reference serum was found to have 2.85 IU (2,185 mIU) of BG-A_{tri} in the sample. This value was used in all subsequent experiments to determine the IU.

One important note: for this approximation to be valid, we assumed that the MFI signal associated with one assay were roughly equivalent to that exhibited by a separate assay. Although this is not true for all Luminex assays as the dynamic range can vary widely, the dynamic range of the IgG and BG-A_{tri} assays using the WHO sample and reference serum, does appear to be similar. Therefore, we felt this assumption was valid.

Table D. Raw Data. MFI values for the serially diluted WHO sample (IgG assay) and the 1:100 dilution of the reference serum (BG-A_{tri} assay) are shown. Results shown are the mean and standard deviation of triplicates.

		WHO	Experiment 1		Experiment 2		Experiment 3	
Assay	D.F.	mIU	MFI	StDev	MFI	StDev	MFI	StDev
IgG	1:100	250	24,216	531	24,817	350	24,488	100
	1:316	79	19,471	380	20,852	639	20,075	463
	1:1,000	25	10,033	285	10,848	68	9,827	283
	1:3,160	7.9	3,990	76	4,625	87	3,948	87
	1:10,000	2.5	1,418	53	1,522	45	1,314	48
	1:100,000	0.3	186	4	199	14	170	1
BG-A_{tri}	1:100		10,445	100	11,465	61	11,927	156

Figure D. Fitted Curve. Data were fitted to a 5-parameter model as described in the Methods section

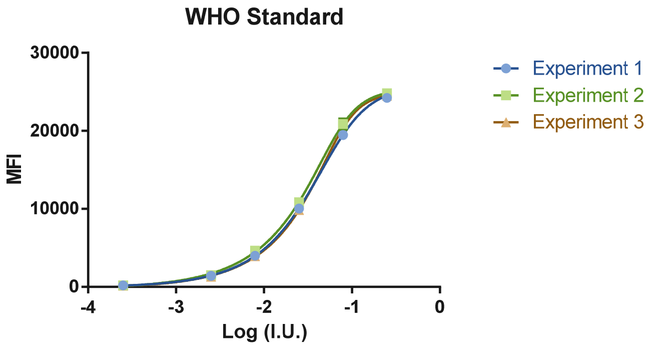

Table E. Interpolated Data.

Sample	Dilution in Linear Range	Mean MFI	Mean I.U.	Standard Deviation	%CV
Reference Serum	1:100	11279	2.85	0.28	9.8%

Appendix A

Standard Operating Procedure

Analyzing Patient Samples for A-tri Antibody Presence

Developed by: MRIGlobal

1470 Treeland Blvd. SE

Palm Bay, FL 32909

Date Approved: April 10, 2015

Introduction

This Standard Operating Procedure (SOP) details an analytical procedure for the qualitative analysis from human serum A-tri IgM antibody presence using Luminex 200.

Contents

Introduction 1

Contents 2

I. Preparation of Phosphate Buffered Saline Solution with 0.1% or 1.0% BSA, 0.02% Tween-20 and 0.05% Sodium Azide and other Luminex-Associated Buffers 3

1. Equipment 3
2. Materials 3
3. Procedures for the Preparation of PBS-TBN 3
4. Procedure for the Preparation of 0.05M MES Coupling Buffer, pH 5.0 5

II. Antigen Detection Assay - Magnetic Bead Coupling, Biotinylation of Detector Antibody 6

1. Equipment 6
2. Materials 6
3. Microsphere Capture Antibody Coupling Procedures 7
4. Biotinylation of Detector Antibody Procedure 9

III. Antigen Detection Assay - Magnetic Bead Procedure for Use with Luminex 200 12

1. Equipment 12
2. Materials 12
3. Procedures 13
4. BioTek Plate Washer Instructions (405 LS) 20
5. Revision/Review History 20

I. Preparation of Phosphate Buffered Saline Solution with 0.1% or 1.0% BSA, 0.02% Tween-20 and 0.05% Sodium Azide and other Luminex-Associated Buffers

1. Equipment

1.1. Stir plate
1.2. Stir bars
1.3. Beaker
1.4. Graduated cylinder
1.5. Appropriate personal protective equipment (PPE)
1.6. Pipette capable of delivering 200µL, and appropriate tips
1.7. Disposable sterile serological pipettes
1.8. Calibrated analytical balance capable of reading 1 gram
1.9. Biological Safety Cabinet (BSC) or clean hood
1.10. Refrigerator (2 to 8°C); temperature monitored daily

2. Materials

2.1. Commercially available Phosphate Buffered Saline (PBS) packets, pH 7.4 (e.g., Sigma Diagnostics P-3813), yielding the following concentrations of these components: 0.01M phosphate buffer saline, 0.138M NaCl, 0.0027M KCl.
2.2. Tween-20, Sigma P-9416
2.3. 5% w/v Sodium Azide (NaN_3), Teknova S0208
2.4. Albumin from bovine serum (BSA), lyophilized powder, RIA Grade, Sigma A-7888
2.5. pH paper capable of measuring pH ~7.4 (i.e., JT Baker (VWR), JT4396-1)
2.6. 0.22µm low protein binding filtration units (45mm), VWR – 28199-812
2.7. Sodium Hydroxide (NaOH), Fisher – SS256-500
2.8. 2[N-Morpholino]ethanesulfonic acid (MES) MES hydrate, Sigma – M2933
2.9. Vacuum trap assembly
2.10. Small weigh boat
2.11. Pipette tips
2.12. 18 Megaohm Water, deionized (DI) Water or MBG Water (interchangeable)

3. Procedures for the preparation of PBS-TBN

3.1. Put on the appropriate PPE (latex or nitrile gloves, laboratory coat and safety glasses).

3.2. This reagent can be prepared in quantities of 1 liter, 2 liters, 3 liters, or 4 liters at a time. See below for quantities of each component.

Total Quantity Prepared	Water	PBS Packets	BSA 0.1% / 1%	Tween-20	5% Sodium Azide
**1 liter**	**990 mL**	**1**	**1 gram / 10 grams**	**200 µL**	**10 mL**
**2 liters**	**1980 mL**	**2**	**2 grams / 20 grams**	**400 µL**	**20 mL**
**3 liters**	**2970 mL**	**3**	**3 grams / 30 grams**	**600 µL**	**30 mL**
**4 liters**	**3960 mL**	**4**	**4 grams / 40 grams**	**800 µL**	**40 mL**

3.3. Measure appropriate amount of water to a graduated cylinder.

3.4. Place a stir bar in the bottom of the beaker and place the beaker on the magnetic stir plate.

3.5. Transfer approximately 70-80% of the total volume of water to the beaker.

3.6. Turn the magnetic stir plate on to a low to medium speed.

3.7. Empty the contents of a commercially prepared PBS packet (appropriate for preparing a 1L solution) into the beaker and allow the solution to mix. Use more PBS packets as needed.

3.8. Stir until the solution is completely mixed.

3.9. Weigh Albumin from bovine serum (BSA) into small weigh boat using a calibrated digital balance. Remember to tare the balance with the small weigh boat first.

3.10. Empty BSA into the beaker and allow solution to mix.

3.11. Stir until the solution is completely mixed.

3.12. Using a pipette, add appropriate amount of Tween-20 to beaker and let dissolve.

3.13. Stir until the solution is completely mixed.

3.14. Add the remainder of the water in the graduated cylinder while the solution is mixing.

3.15. Confirm that pH is still ~7.4 using pH paper.

3.16. Remove beaker from stir plate and put in BSC. Using a graduated cylinder or serological pipette, the appropriate amount of 5% Sodium Azide (NaN_3) to beaker. Mix thoroughly.

3.16.1. Store at 2 to 8°C.

3.17. Label the solution with the following: PBS-TBN, Lot #, storage conditions, expiration date (1 year after preparation), and initialed by the preparer. Lab notebook contains preparer's signature.

4. Procedure for the preparation of 0.05M MES Coupling Buffer, pH 5.0

4.1. Put on the appropriate PPE (latex or nitrile gloves, laboratory coat and safety glasses).

4.2. Measure 250mL of water to a graduated cylinder

4.3. Place a stir bar in the bottom of the beaker and place the beaker on the magnetic stir plate.

4.4. Transfer the total volume of water to the beaker.

4.5. Turn the magnetic stir plate on to a low to medium speed.

4.6. Weigh approximately 2.44grams of 2[N-Morpholino]ethanesulfonic acid (MES) into small weigh boat using a calibrated digital balance. Remember to tare the balance with the small weigh boat first.

4.7. Empty MES into the beaker and allow solution to mix.

4.8. Stir until the solution is completely mixed.

4.9. Using a pipette, add 5 drops of 5N NaOH to beaker and let dissolve.

4.10. Stir until the solution is completely mixed.

4.11. Confirm that pH is ~5.0 using pH paper.

4.11.1. If the pH indicator strip indicates that the solution is too acidic, additional drops of NaOH may be added until the pH reaches 5.0. Check the pH after each additional drop of NaOH is added to reach a pH of ~5.0.

4.12. Once the solution is thoroughly mixed, filter sterilize the solution using a 0.22μm filter. The following procedure should be followed:

4.12.1. Attach the filter tubing adaptor to the filter unit at the spout.

4.12.2. Attach one end of a piece of tubing to the filter tubing adaptor and the other end to a vacuum line or vacuum pump. Note: The vacuum lines must be HEPA-filtered with a containment trap to prevent filtered material from entering vacuum pump.

4.12.3. Remove the lid on the filter unit and turn on the vacuum.

4.12.4. Once the solution passes through the filter completely, turn off the vacuum.

4.12.5. Once all filtration has been completed, move the filtration unit to the BSC. Open the package for the cap using sterile handling procedures. Do not touch the inside of the cap.

4.12.6. Remove the pre-filter unit by twisting it off and immediately cap the post filter unit using the appropriate cap.

4.12.7. Discard the pre-filter unit.

4.13. Store at 2° to 8°C.

4.14. Label the solution with the following: 0.05M MES Coupling Buffer, pH 5.0, Lot#, storage conditions, expiration date (1 year after preparation) and initialed by the preparer. Lab notebook contains preparer's signature.

II. Antigen Detection Assay - Magnetic Bead Coupling, Biotinylation of Detector Antibody

1. Equipment

1.1. Pipettes and appropriate tips

1.2. Magnetic Tube Separators (Luminex – CN-0288-01)

1.3. Tube Rotator (VWR – 13916-822)

1.4. Vortex

1.5. Tube racks

1.6. 1.5mL USA Scientific microcentrifuge tubes, (USA Scientific 1415-2500)

1.7. 15mL conical tubes

1.8. Refrigerator (2 to 8°C), temperature monitored daily

1.9. Biological Safety Cabinet (BSC)

1.10. Centrifuge capable of holding 15mL conical tubes and spinning at 1,000rcf

1.11. Appropriate Personal Protective Equipment (lab coats, safety glasses and gloves)

2. Materials

2.1. Luminex magnetic microspheres (Luminex – MC10013-01, MC10030-01, MC10045-01, MC10073-01)

2.2. Luminex Coupling Kit (Contains Activation, Wash buffers, EDC and Sulfo-NHS), (Luminex - 40-50016)

2.3. Extended Fine Tip Transfer Pipet (Fisher Scientific – 13-711-29)

2.4. EDC Reagent (10mg Vial) (Pierce – 77149)

2.5. EZ-Link Sulfo-NHS-Biotin (Pierce – 21326)

2.6. 0.05mM MES Coupling buffer (pH 5.0)

2.7. MBG or DI, 18 Megaohm water (interchangeable)

2.8. Capture antigen for coupling: Blood Group A NGP – 6 atom spacer BSA (V-Labs – NGP6305), Galα1-3Galβ1-4Glc-3 atom spacer BSA (V-Labs – NGP0330), Albumin from bovine serum (Sigma – A7888), IgG human serum (Sigma – I4506)

2.9. Phosphate Buffered Saline solution (PBS) with 0.1% BSA, 0.02% Tween-20 and 0.05% Sodium Azide (TBN), pH 7.4

2.10. Detection antibody: AffiniPure Goat Anti-Human IgM, $Fc_{5\mu}$ Fragment Specific (Jackson ImmunoResearch – 109-005-043)

2.11. Zeba desalting columns, 2mL (Pierce - 89890)

2.12. Sulfo-NHS, No-Weigh Format (Pierce – 24520)

3. Microsphere-Capture Antibody Coupling Procedures

3.1. Remove kit from the refrigerator and allow it to equilibrate to room temperature (at least 30 minutes). During the procedure, minimize light exposure on the beads.

3.2. If needed, reconstitute lyophilized antigen proteins with 1mL water and vortex, final concentration 1mg/mL and store at -20°C.

3.3. Select appropriate bead region and capture antibody for assay(s):

Assay	Bead Region	Capture Antigen
A-tri	13	Blood Group A NGP – 6 atom spacer BSA
Galili	30	Galα1-3Galβ1-4Glc-3 atom spacer BSA
BSA	45	Albumin from bovine serum
IgG	73	IgG human serum

3.4. Calculate the volume of stock microspheres and capture antigen needed (No bead prep should exceed 5 x10^6 beads/mL)

3.4.1. Microspheres:

3.4.1.1. Volume of stock needed = (# of beads to be coupled ÷ stock concentration)

3.4.1.2. Ex. Volume stock needed = (5 x10^6 beads ÷ 12.5 x10^6 beads/mL) = 0.4mL or 400µL

3.4.2. Capture Antigen:

3.4.2.1. Volume of Stock antigen needed = (#of beads to be coupled) (5µg/ $1x10^6$ beads) ÷ (stock capture Ab concentration)

3.4.2.2. Ex. Volume of stock antigen = ($5x10^6$ beads) (5µg/ $1x10^6$ beads) ÷ 1 mg/mL = 25µL

3.5. Resuspend the stock microspheres.

3.5.1. If using a 1mL stock microsphere vial, vortex the stock microsphere vial for 10 seconds to disperse the microspheres. Alternatively, the microsphere vials can be rotated on a rotator for 15 minutes.

3.6. Dispense the calculated volume of stock microspheres into a clean 1.5mL USA Scientific tube.

3.7. Place reaction tube inside the magnetic tube separator for at least 1 minute.

3.8. With the tube still in the separator, remove the supernatant with an extended fine tip transfer pipet, being careful to not disturb the beads. Remove tube from magnetic separator.

3.9. Add 100µL of distilled water to the tube. Vortex for ~20 seconds.

3.10. Place reaction tube inside the magnetic tube separator for at least 1 minute.

3.11. With the tube still in the separator, remove the supernatant with an extended fine tip transfer pipet, being careful to not disturb the beads. Remove tube from magnetic separator.

3.12. Add 80µL of Activation buffer (provided by manufacturer in Luminex coupling kit; pH not listed) to the tube. Vortex for ~20 seconds.

3.13. Add 10µL of Sulfo-NHS (from kit) to the tube and gently vortex. If using supplemental Sulfo-NHS (Sulfo-NHS, No-Weigh Format), add 40µL of water to the bullet containing 2mg of Sulfo-NHS. Vortex thoroughly.

3.14. Add 200µL of distilled water to 10mg vial of EDC.

3.15. Please note that EDC will immediately degrade upon exposure to light and moisture. Therefore, EDC should be prepared and used IMMEDIATELY, and discarded after use.

3.16. Vortex EDC preparation for ~10 sec.

3.17. Add 10µL of EDC to the microspheres. Vortex gently.

3.18. Incubate at room temperature for 20 minutes, being sure to protect tubes from light. Gently mixing by vortex at 10 minute intervals.

3.19. Place reaction tube inside the magnetic tube separator for at least 1 minute.

3.20. With the tube still in the separator, remove the supernatant with an extended fine tip transfer pipet, being careful to not disturb the beads. Remove tube from magnetic separator.

3.21. Add 250µL of 50mM MES, pH 5.0 to the tube. Vortex for ~20 seconds.

3.22. Repeat 3.19-3.21 for a total of 2 washes in 50mM MES, pH 5.0.

3.23. Remove tube from magnetic separator and resuspend the activated and washed microspheres in 100µL of 50mM MES, pH 5.0 and vortex ~20 seconds.

3.24. Vortex stock capture antigen. Add 5µg protein/$1x10^6$ beads (as calculated in 3.4.2) to the resuspended microspheres.

3.25. Bring total volume to 500µL with 50mM MES, pH 5.0. 500µL (Total Volume)- 100µL (MES buffer already added)- 25µL (Protein added) = 375µL MES Buffer added

3.26. Vortex thoroughly.

3.27. Parafilm tube and incubate at room temperature for 2 hours using a tube rotator, making sure to protect tubes from light by wrapping rotator in foil.

3.28. Wash the microspheres:

3.28.1. Place reaction tube inside the magnetic tube separator for at least 1 minute.

3.28.2. With the tube still in the separator, remove the supernatant with a transfer pipet, being careful not to disturb the beads. Remove tube from magnetic separator.

3.28.3. Add 500µL of PBS-TBN to the tube.

3.28.4. Vortex for ~20 seconds.

3.28.5. Place reaction tube inside the magnetic tube separator for at least 1 minute.

3.28.6. With the tube still in the separator, remove the supernatant with a transfer pipet, being careful not to disturb the beads. Remove tube from magnetic separator.

3.28.7. Add 1mL of PBS-TBN to the tube. Vortex for ~20 seconds.

3.28.8. Repeat steps 3.28.5-3.28.7 for a total of 2 washes.

3.29. Place the reaction tube in the magnetic separator for at least 1 minute.

3.30. With the tube still in the separator, remove the supernatant with a transfer pipet, being careful not to disturb the beads.

3.31. Resuspend the microspheres with 1mL of PBS-TBN.

3.32. Store at 2 to 8°C, protected from light. Microspheres need to be stored overnight before use.

4. Biotinylation of Detector Antibody Procedure

4.1. Select appropriate detector antibody for assay(s):

Assay	Detector Antibody
All	AffiniPure Goat Anti-Human IgM, Fc5µ Fragment Specific

4.2. Calculations:

4.2.1. Determine the amount of stock detection antibody needed to obtain a theoretical yield of 1mg/mL at a final volume of 200µL to 400µL.

4.2.1.1. Example: 200µL prep will be made using a stock dAb of 2 mg/mL: (x)(2 mg/mL) = (1mg/mL)(0.200mL) ; x = 100 µL of stock dAb

4.2.2. Determine the amount of EZ-Link Sulfo-NHS-Biotin that is needed for the reaction: 50µL for a 200µL prep, 100µL for a 400µL prep.

4.2.3. Calculate PBS-TBN stacker volume needed to achieve the final volume by subtracting the dAb and Biotin volumes from the final desired volume.

4.2.3.1.Example: 200µl – (50µL + 100 µL) = 50µL of buffer stacker

4.3. Prepare EZ-Link Sulfo-NHS-Biotin by adding 200µL of PBS-TBN buffer to one 1mg EZ-Link Sulfo-NHS-Biotin bullet.

4.4. Parafilm the Biotin bullet and vortex for ~10 seconds.

4.5. Vortex detection antibody for ~10 seconds.

4.6. Pulse centrifuge detector antibody to ensure it is collected at the bottom of the tube. Pipet up and down and disperse the calculated volume of dAb into a clean 1.5mL USA Scientific tube.

4.7. Disperse the calculated volume of Biotin into the reaction tube.

4.8. Incubate reaction tube at room temperature for 30 minutes while protected from light (wrap tube with foil).

4.9. Remove the breakable tab from the bottom of the Zeba de-salting spin column.

4.10. Place spin column into a clean 15mL collection conical.

4.11. Mark one side of the spin column tube with a marker or piece of tape.

4.12. Centrifuge de-salting spin column at 1,000rcf for 2 minutes; making sure that the column is positioned so that the mark is facing to the outside. Please note that a balance tube may be needed if preparing a single dAb biotinylation preparation.

4.13. Remove the column from the centrifuge and empty the contents of the collection tube.

4.14. Wash the de-salting spin column:

4.14.1. Return column to the collection conical.

4.14.2. Wash with 1mL of PBS-TBN buffer to the spin column.

4.14.3. Centrifuge the column for 2 minutes at 1,000rcf making sure that the mark on the de-salting spin column is facing outward.

4.14.4. Empty the contents of the collection tube.

4.15. Repeat step 4.14 twice more for a total of three washes.

4.16. Place de-salting spin column into a labeled and clean 15mL collection conical.

4.17. Disperse the contents of the reaction tube into the de-salting spin column making sure that the contents are placed directly onto the column and not the walls.

4.18. After the sample has fully absorbed into the resin bed, add the calculated PBS-TBN buffer stacker to the column.

4.19. Centrifuge the column for 2 minutes at 1,000rcf making sure that the mark on the de-salting spin column is facing outward.

4.20. Determine the recovery concentration by using 2µL of the preparation on a Nanodrop set to “protein A280” and sample type “IgG”.

4.21. Be sure to run 2µL of water to wet the Nanodrop column and 2µL of PBS-TBN to blank the Nanodrop before reading the sample.

4.22. If preparing multiple preps of the same agent detection antibody, the preps can be pooled together to form a single lot. However, each preparation needs to be analyzed on the Nanodrop prior to combining and once more after being pooled.

III. Antigen Detection Assay -

Magnetic Bead Procedure for Use with Luminex 200

1. Equipment
   - 1.1. Luminex 200 analyzer: A computer loaded with Luminex xPONENT software version 3.1 Build 971 (or newer) coupled to an 200 analyzer and SD system (Source: Luminex)
   - 1.2. Pipettes (calibrated and certified) and appropriate tips
   - 1.3. BioTek 96-probe Plate Washer (405 LS) (or equivalent plate washer) with Sample Analysis Wash and Sample Analysis Dispense Programs loaded onto the instrument
   - 1.4. Vortex
   - 1.5. Tube racks
   - 1.6. Refrigerator (2 to 8°C), temperature recorded daily
   - 1.7. Freezer (-10 to -25°C), temperature recorded daily
   - 1.8. Biological Safety Cabinet
   - 1.9. Magnetic Plate Separator (Luminex, CN-0269-01, center capture) OR BioTek 405LS Plate Washer
   - 1.10. Titer Plate Shaker (i.e., Thermo Scientific, 4625Q orbital 96 well plate shaker or similar)
   - 1.11. Luminex dark plate cover
   - 1.12. Appropriate Personal Protective Equipment (lab coats, safety glasses and gloves)
2. Materials
   - 2.1. Phosphate Buffered Saline solution (PBS) with 0.1% BSA, 0.02% Tween-20 and 0.05% Sodium Azide (TBN) prepared per Section I.3, store at 2 to 8°C (store at room temperature for plate washer). (PBS-TBN 0.1%), pH 7.4
   - 2.2. Phosphate Buffered Saline solution (PBS) with 1% BSA, 0.02% Tween-20 and 0.05% Sodium Azide (TBN) prepared per Section I.3, store at 2 to 8°C. (PBS-TBN 1%), pH 7.4
   - 2.3. Luminex Sheath Fluid; expiration date supplied by manufacturer, store at room temperature
   - 2.4. Human Serum (Male) – BioreclamationIVT – HMSRM-M
   - 2.5. WHO Rheumatoid Factor Reference Serum (NIBSC, Part Number W1066)

2.6. Liquicheck Rheumatoid Factor Control, Level 1 (BioRad, 501)

2.7. PBS

2.8. Coupled beads and capture antigens, LIGHT SENSITIVE, remove just before use.

2.9. Biotinylated detector antibody (see Biotinylation of Detector Antibody Procedure in Section II.4 above)

2.10. Streptavidin – Phycoerythrin conjugate. LIGHT SENSITIVE, remove from box just before use (Moss, Inc, SAPE-001; see product sheet for expiration date)

2.11. Reagent Reservoirs

2.12. Costar White Round Bottom Plates

2.13. 1.5mL USA Scientific microcentrifuge tubes, (USA Scientific 1415-2500)

3. Procedures

3.1. Run the System Initialization on the Luminex 200 instrument (Laser warm-up and fluidics). Make sure the Luminex SD is turned on and has sufficient sheath fluid during start up prior to starting System Initialization. Check the waste container level.

3.2. After System Initialization is complete, run the Performance Verification program on the Luminex 200 instrument. Once a week, sonicate the probe for 20 minutes and readjust the probe height. Run the Calibration/Verification in lieu of the Performance Verification.

3.3. The plate layout is shown below. Up to 19 samples can be run per plate. There are four external controls and a standard curve (used to calculate sample concentration for the Atri and Galili assays in mIU). These are briefly described in the bullet points below. Preparation of the standard/reference curve, samples and controls is detailed in the sections below.

- Blank (buffer only)
- Negative Serum Control 1:50; BioRad control, Level 1 for this (very low Atri)
- High Positive Serum Control 1:50; pooled Male Type B serum, same as that used for the standard or reference curve (high Atri)
- Low Positive Serum Control 1:50 (WHO control serum; equivalent to a low Atri)
- Standard or Reference curve; pooled Male Type B serum; same as high positive serum control)

	1	2	3	4	5	6	7	8	9	10	11	12
A	Blank	Reference Serum 1:10935	Reference Serum 1:10935	Reference Serum 1:10935	Sample 2	Sample 2	Sample 2	Sample 10	Sample 10	Sample 10	Sample 18	Blank
B	Neg Serum 1:50	Reference Serum 1:3645	Reference Serum 1:3645	Reference Serum 1:3645	Sample 3	Sample 3	Sample 3	Sample 11	Sample 11	Sample 11	Sample 18	Neg Serum 1:50
C	High Pos Serum 1:50	Reference Serum 1:1215	Reference Serum 1:1215	Reference Serum 1:1215	Sample 4	Sample 4	Sample 4	Sample 12	Sample 12	Sample 12	Sample 18	High Pos Serum 1:50
D	Low Pos Serum 1:50	Reference Serum 1:405	Reference Serum 1:405	Reference Serum 1:405	Sample 5	Sample 5	Sample 5	Sample 13	Sample 13	Sample 13	Sample 19	Low Pos Serum 1:50
E	Blank	Reference Serum 1:135	Reference Serum 1:135	Reference Serum 1:135	Sample 6	Sample 6	Sample 6	Sample 14	Sample 14	Sample 14	Sample 19	Blank
F	Neg Serum 1:50	Reference Serum 1:45	Reference Serum 1:45	Reference Serum 1:45	Sample 7	Sample 7	Sample 7	Sample 15	Sample 15	Sample 15	Sample 19	Neg Serum 1:50
G	High Pos Serum 1:50	Reference Serum 1:15	Reference Serum 1:15	Reference Serum 1:15	Sample 8	Sample 8	Sample 8	Sample 16	Sample 16	Sample 16		High Pos Serum 1:50
H	Low Pos Serum 1:50	Sample 1	Sample 1	Sample 1	Sample 9	Sample 9	Sample 9	Sample 17	Sample 17	Sample 17		Low Pos Serum 1:50

3.4. A Reference Curve using pooled Male Type B Reference Serum will be run on each plate (Serum dilutions and Blanks in triplicate):

3.4.1. Dilute the stock standard serum to a 1:15 dilution using PBS as the serum diluent and perform serial 1:3 dilutions of the 1:15 standard serum to a final concentration of 9.15e-5 (1:10,935).

Dilution Tube	Volume of PBS Buffer	Volume of Standard Serum	Concentration (1/D.F.)
1:15	466.7 µL	33.3 µL (1x)	0.066666667
1:45	333.3 µL	166.7 µL (1:15)	0.022222222
1:135	333.3 µL	166.7 µL (1:45)	0.007407407
1:405	333.3 µL	166.7 µL (1:135)	0.002469136
1:1,215	333.3 µL	166.7 µL (1:405)	0.000823045
1:3,645	333.3 µL	166.7 µL (1:1,215)	0.000274348
1:10,935	333.3 µL	166.7 µL (1:3,645)	9.14495E-05

3.4.2 Add 50 µL of PBS Buffer to the Blank wells.

3.4.3 Vortex and add 50 µL of the serum dilution preparations to their respective wells working from a low to high concentration, as per plate layout.

3.5. All handling of samples and controls must be performed in the biosafety cabinet (BSC). It is imperative that the plate remain flat AT ALL TIMES.

3.5.1. Sample and Control serum dilutions:

3.5.1.1 Serum samples will be run in triplicate on the plate at 1:50 dilution.

3.5.1.2 Three serum controls at 1:50 will be run on each plate: a very low A-tri (from Bio-Rad; Negative Control), a high A-tri (the pooled Type B reference serum used in the standard curve; High Positive Control), and one control in the linear range (from WHO; Low Positive Control). Consult the plate layout for replicate number of each control.

3.5.1.3 Dilute the serum or control sample to a 1:50 dilution using PBS buffer as the sample diluent.

An example of the dilution scheme is:

Dilution Tube	Volume of PBS Buffer	Volume of Serum Sample	Concentration (1/D.F.)
1:50	490 µL	10 µL (Stock Sample)	0.02

3.5.2. Consult the plate layout for placement of samples and controls on each plate and add 50 µL of each sample or control to the appropriate wells.

3.6. Remove bead/ capture antigen mix for each bead region. Vortex the bead/capture antigen for 30 seconds. Prepare bead solution for plate by following dilution information: (Multiplex: Each bead region is at 50 beads/µL). The bead solution needs to be brought to volume with PBS-TBN 1%. Vortex the solution for 30 seconds. If a multi-channel pipette is to be used, pour the bead solution into a reagent reservoir. Pipette 50µL of bead solution into each well in use, according to the plate layout. Note: if the bead solution sits idle for any length of time, the solution must be mixed thoroughly again.

3.6.1 Example (48 reactions) – Each bead region has a stock concentration 5000beads/ul that needs to be diluted to 50beads/ul. This will give 2500beads/well.

$(x)(5000\ beads/ul) = (50\ beads/uL)(2400ul) => x = 24\mu L$ of stock microspheres. Vortex and added 24µL of each bead region to 2304µL PBS-TBN 1%.

3.7. Once all controls and samples have been loaded into appropriate wells as well as the multiplexed bead mix, cover plate with a Luminex dark plate cover to protect the samples from light.

3.8. Carefully place plate on the titer plate shaker, ensure the plate is secure, and shake the plate for 1 hour at 800rpm. If you are manually pipetting supernatant off of the plate, continue to 3.9. If you are using the BioTek plate washer (405 LS) (or BioTek EL50), follow wash instructions in 4.1.

3.9. Carefully remove plate from the titer plate shaker and place on magnetic plate separator. Remove plate cover and lock magnet brackets on plate. (Note: Hold plate while putting brackets on plate to keep plate from tipping). Leave plate on magnet for at least 1 minute.

3.10. Remove supernatant (approximately 100µL) from each well, being careful not to disturb the bead pellet (may be visible) at the bottom of the plate. Supernatant should be pulled off by angling the pipette tips to the side of the well. (Note: there will be some volume left behind in the wells. Make an effort to remove all of the supernatant. Repeatedly going over the wells will increase the likelihood of beads being removed. However, if less than half of the recommended volume is removed, the analyst should attempt to remove additional supernatant from those specific wells only.)

3.11. Remove plate from magnetic plate separator. Add 100µL of PBS-TBN 0.1% to each reaction well to wash the beads.

3.12. Place plate on magnetic plate separator (locking clips) for at least 1 minute.

3.13. Remove supernatant (approximately 100µL) from each well, being careful not to disturb the bead pellet at the bottom of the plate. Supernatant should be pulled off by angling the pipette tips to the side of the well. (Note: there will be some volume left behind in the wells. Make an effort to remove all of the supernatant. Repeatedly going over the wells will increase the likelihood of beads being removed. However, if less than half of the recommended volume is removed, the analyst should attempt to remove additional supernatant from those specific wells only.)

3.14. Repeat steps 3.11 through 3.13 again, for a total of 2 washes.

3.15. Obtain the detector antibody for plate. Vortex the solution for 30 seconds. Prepare detector antibody solution for plate by following dilution information: (Multiplex: 4µg/mL). Note: This reagent will have varying concentrations based on results of biotinylation. The detector antibody solution needs to brought to volume with PBS-TBN 1%. Vortex the solution thoroughly. Using a multi-channel pipette, pour into reagent reservoir. Pipette 100µL of detector antibody solution into each well in use, according to the plate layout on the 200. A single channel pipette can also be used if desired. Note: if the detector antibody solution sits idle for more than 1 minute, the solution must be mixed thoroughly again. Visually inspect plate to ensure that each well has detector antibody mix. Cover plate with Luminex dark plate cover to protect the samples from the light.

3.16. Carefully place plate on the titer plate shaker, ensure the plate is secure, and shake the plate for 30 minutes at 800rpm. If you are manually pipetting supernatant off of the plate, continue to 3.17. If you are using the BioTek plate washer (405 LS), follow wash instructions in 4.1.

3.17. Carefully remove plate from the titer plate shaker and place on magnetic plate separator. Remove plate cover and lock magnet brackets on plate. (Note: Hold plate while putting brackets on plate to keep plate from tipping) Leave plate on magnet for at least 1 minute.

3.18. Remove supernatant (approximately 100µL) from each well, being careful not to disturb the bead pellet at the bottom of the plate. Supernatant should be pulled off by angling the pipette tips to the side of the well. (Note: there will be some volume left behind in the wells. Make an effort to remove all of the supernatant. Repeatedly going over the wells will increase the likelihood of beads being removed. However, if less than half of the recommended volume is removed, the analyst should attempt to remove additional supernatant from those specific wells only.)

3.19. Remove plate from magnetic plate separator. Add 100µL of PBS-TBN 0.1% to each reaction well to wash the beads.

3.20. Place plate on magnetic plate separator (locking clips) for at least 1 minute.

3.21. Repeat steps 3.18 through 3.20 for a total of 2 washes.

3.22. Obtain the Streptavidin – Phycoerythrin conjugate (SAPE). Vortex the tube. Prepare SAPE solution for plate. Bring the SAPE to volume with PBS-TBN 1% so that the final concentration of SAPE is at 4µg/mL; 10 mL will be needed for a full plate. Vortex the solution for 30 seconds. If using a multi-channel pipette, pour into reagent reservoir. Pipette 100µL of SAPE solution into each well in use, according to the plate layout. Note: if the SAPE solution sits idle for more than 10 seconds, the solution must be mixed thoroughly again. Visually inspect plate to ensure that each well has SAPE solution. Cover plate with Luminex dark plate cover to protect the samples from the light.

3.23. Carefully place plate on the titer plate shaker, ensure the plate is secure, and shake the plate for 30 minutes at 800rpm. If you are manually pipetting supernatant off of the plate, continue to 3.24. If you are using the BioTek plate washer (405 LS), follow wash instructions in 4.2.

3.24. Carefully remove plate from the titer plate shaker and place on magnetic plate separator. Remove plate cover and lock magnet brackets on plate. (Note: Hold plate while putting brackets on plate to keep plate from tipping). Leave plate on magnet for at least 1 minute.

3.25. Remove supernatant (approximately 100µL) from each well, being careful not to disturb the bead pellet at the bottom of the plate. Supernatant should be pulled off by angling the pipette tips to the side of the well. (Note: there will be some volume left behind in the wells. Make an effort to remove all of the supernatant. Repeatedly going over the wells will increase the likelihood of beads being removed. However, if less than half of the recommended volume is removed, the analyst should attempt to remove additional supernatant from those specific wells only.)

3.26. Remove plate from magnetic plate separator. Add 100µL of PBS-TBN 0.1% to each reaction well to wash the beads.

3.27. Place plate on magnetic plate separator (locking clips) for at least 1 minute.

3.28. Repeat steps 3.25 through 3.27 for a total of 2 washes.

3.29. Add 100µL of PBS-TBN 0.1% to each reaction well.

3.30. Carefully place plate on the titer plate shaker, ensure the plate is secure, and cover plate with Luminex dark plate cover to protect samples from the light. Shake the plate for at least 30 seconds at 800rpm. The plate must be placed in the instrument immediately after shaking. If there is a delay, the plate must be shaken again for at least 30 seconds.

3.31. Click 'Batches' tab in Luminex xPonent software.

3.32. Click 'Create New Batch from Existing Protocol' option, step 1 of 2 will open.

3.33. Enter a name for the plate in the 'Batch Name' field.

3.34. Select 'Gildersleeve – multi' from the list of protocols. Click 'Next Step' in the bottom right of the step 1 window.

3.35. Highlight all control and sample wells as per plate layout and click 'Unknown' to label the sample wells with appropriate bead regions. (Note: Do not select 'Background' or 'Control' options as sample type.) Individually name all samples or import a file with sample names.

3.36. Highlight wells A2 – A12 and E1 – E12 (as appropriate to plate layout, do not select out past the samples on the plate layout). Under the 'Commands' section, click on the dropdown menu and select 'Wash'. Click the 'Before Well' icon under the dropdown menu.

3.37. Click 'Save'.

3.38. Click "Eject" on the 200 instrument toolbar to eject the plate tray.

3.39. Place the prepared plate in the plate tray with well A1 as indicated on plate tray. Load water into the black off plate reservoir. Click Retract on the toolbar to load plate into the instrument.

3.40. To read a plate, perform the following:

3.40.1. Verify the information on both plate layouts is correct. Utilize the 'Edit' function to make any necessary changes.

3.40.2. Click 'Run' on the toolbar to start the plate reading process.

3.40.3. Click 'Eject' from the toolbar to eject the plate tray when the plate is completed.

3.40.4. Run the 'Stringent Clean with NaOH' after each plate.

3.40.5. Export the completed run to a .csv file by selecting the "Results" tab. Select "Saved Batches" in the submenu and select the appropriate plate to be exported. Select "Exp Results" and export the file to the desired location.

3.41. Data Analysis and Acceptance Criteria

3.41.1. Check all bead counts for each bead region, if any are below 35, then data from that well cannot be used.

3.41.2. Assay tolerance ranges (External Serum Controls) for Atri and Galili assays are below. BSA and IgG assays are excluded from criteria (mIU can't be determined). Plates with values outside these ranges must be repeated.

Tolerance Range Atri mIU	Mean	SD	Minimum	Maximum
**Neg Serum 1:50**	12.6	11.0	**0**	**47**
**High Pos Serum 1:50**	2,799	418	**1,545**	**4,053**
**Low Pos Serum 1:50**	40.8	6.6	**20**	**61**

Tolerance Range Galili mIU	Mean	SD	Minimum	Maximum
**Neg Serum 1:50**	46.3	6.1	**28**	**65**
**High Pos Serum 1:50**	3,127	392	**1,951**	**4,303**
**Low Pos Serum 1:50**	59.5	7.1	**38**	**81**

3.41.3. Internal Controls

3.41.3.1. IgG assay. For samples that are determined to be above the threshold (predicted responders) for Atri (see Section 3.41.5). Divide the sample IgG assay (bead region 73) MFI value by the mean Negative Serum Control MFI value (bead region 73, IgG assay). Samples with 0.75 or greater value are flagged and will be tested to determine their Rf levels in IU.

3.41.3.1.1. To determine Rf levels on flagged samples: Dilute sample 1:1,000. Run sample in triplicate using the WHO serum as the standard curve (Dilution range is 1:100 – 1:100,000; this corresponds to 250 – 0.25 mIU). Interpolate sample MFI values for the IgG assay (bead region 73) to the curve to obtain the concentration in IU. Samples with Rf values above 15 IU are considered above the normal range.

3.41.3.2. Galili assay. Interpolate values for the Galili assay (bead region 30) as described in section 3.41.4. Calculate the amount in mIU. Samples with a value greater than 0.25 mIU are considered positive.

3.41.3.3. BSA assay. Use as an internal background signal for each well to account for variability in BSA background in each sample. Subtract the BSA assay MFI signal (bead region 45) from the Atri MFI signal (bead region 13) from the SAME well. Then analyze data as described in Section 3.41.4 below.

3.41.4. Regression analysis using standard curve. Fit data to standard curve and interpolate samples. Data for Atri and Galili assays are fitted using nonlinear regression analysis (i.e., GraphPad Prism or similar software). A five parameter logistic curve (Richard's model) is used, since these curves are asymmetric around the midpoint. MFI values for each assay are interpolated against the fitted curve of the same assay. Bead region 13 is used for Atri and bead region 30 is used for

Galili.

3.41.4.1. Units: standard reference serum (pooled male type B serum used in the standard curve) in mIU. The amount of Atri and Galili present in the reference serum in mIU will be determined with every new lot of pooled Male Type B serum, using the WHO reference serum; the current stock is at 2.85 IU (2,850 mIU) for Atri and 3.22 IU (3,220 mIU) for Galili.

3.41.4.2. The resulting interpolated values are multiplied by 50 (the dilution factor) to obtain the final amount in mIU. Refer to Section 3.41.3.2 for Galili data interpretation and Section 3.41.5 for Atri.

3.41.5. Responder/Non-Responder Threshold.

3.41.5. The threshold will be determined as more data is collected.

4. BioTek Plate Washer Instructions (405 LS) – These instructions are dependent on model of plate washer and may need to be modified based on what the lab is using.

4.1. After the serum incubation, transfer the plate to BSC with plate washer. Carefully place plate on magnet and remove dark plate cover. At the main menu, select 'RUN' on the screen. Select protocol '17' ('SA WASH') and hit enter. Hit start. The program will run through the 2 wash steps. When the protocol is done, put the dark plate cover on the plate and transfer it back to the procedure hood. Resume procedure at 3.15. After the detector antibody procedure, resume the procedure at 3.22.

4.2. After the SAPE incubation, run the 'SA WASH' protocol as outlined in 4.1. When the protocol is done, leave the plate on the magnet. Select protocol '18' ('SA DISP') and hit enter. Hit start. The protocol will dispense the final 100µL of PBS-TBN 0.1% to each reaction well. When the protocol is done, put the dark plate cover on the plate and transfer it back to the procedure hood. Continue with procedure at 3.29.
